# N6-methyladenosine (m^6^A) modification of TXNIP in 3′UTR instigates abdominal aorta aneurysm in mice

**DOI:** 10.1016/j.isci.2026.114630

**Published:** 2026-01-07

**Authors:** Fransky Hantelys, Wenfeng Yin, Ming Hui Zou

**Affiliations:** 1 Department of Endocrinology and Metabolism, Tianjin Medical University General Hospital, 154 Anshan Road, Tianjin 300052, China; 2 Center for Molecular and Translational Medicine, Georgia State University, Atlanta, GA 30303, USA

**Keywords:** Biochemistry, Molecular biology

## Abstract

The thioredoxin-interacting protein (TXNIP) pathway is a central regulator of oxidative stress and contributes to vascular pathology. Here, we define how stress-responsive mRNA methylation controls TXNIP expression and drives abdominal aortic aneurysm (AAA). In angiotensin II (AngII)-infused *ApoE*^−/−^ mice, TXNIP was markedly elevated in vascular smooth muscle cells (VSMCs), as confirmed by histological, protein, and transcript analyses. VSMC-specific TXNIP deletion (*ApoE*^−/−^*TXNIP*^*SM−/−*^) significantly reduced AAA incidence, aortic remodeling, and elastic fiber degradation, establishing its essential role in disease progression. Mechanistic studies revealed that elevated m^6^A methylation, catalyzed by METTL3, promoted TXNIP translation via YTHDF1 binding to m^6^A sites within the 3′ untranslated region (UTR), whereas YTHDF2 downregulation in AAA stabilized TXNIP transcripts. TXNIP translation also proceeded through a cap-independent process enhanced by mTOR inhibition. These findings identify an integrated m^6^A-dependent regulatory program governing TXNIP expression and highlight therapeutic opportunities for targeting AAA progression.

## Highlights

• TXNIP upregulation induces AAA formation
• Silencing YTHDF2 increased *TXNIP* mRNA stability
• TXNIP translation is YTHDF1-binding dependent
• m^6^A within 3′UTR of *TXNIP* mRNA promotes its cap-independent translation

## Graphical abstract

**Figure:**
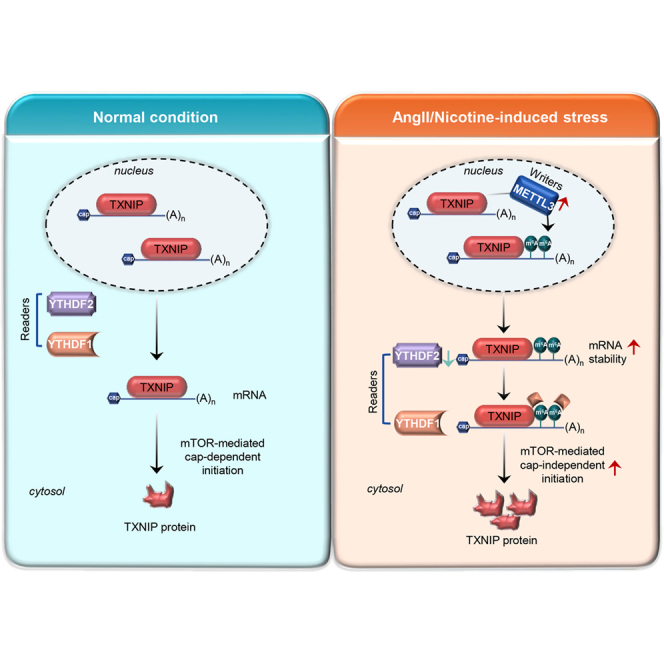

## Introduction

An abdominal aortic aneurysm (AAA) is a localized enlargement of the abdominal aorta, typically exceeding 3 cm in diameter.^1^ This condition arises due to the weakening of the arterial wall, often linked to atherosclerosis, chronic hypertension, smoking, or genetic predisposition.^2^^,^^3^^,^^4^ If left untreated, AAA can progress to rupture, resulting in catastrophic bleeding with a mortality rate exceeding 80%.^5^ Currently, surgical intervention remains the only effective treatment for AAA.^1^^,^^6^ Understanding how AAA risk factors compromise the aortic wall is essential for developing novel therapeutic approaches for this life-threatening condition.

Vascular smooth muscle cells (VSMCs), the primary cellular component of the aortic wall, play a pivotal role in AAA development and progression.^7^ These cells maintain vascular integrity by producing extracellular matrix (ECM) components such as collagen and elastin, which provide structural support. In AAA, VSMCs undergo dysfunction and apoptosis, leading to ECM degradation and weakening of the aortic wall. This process is driven by oxidative stress, inflammation, and imbalances in proteolytic enzymes (e.g., matrix metalloproteinases).^8^^,^^9^ VSMC loss disrupts aortic repair mechanisms, accelerating aneurysm progression and increasing the risk of rupture.^10^ Furthermore, VSMCs adopt a synthetic phenotype, producing inflammatory cytokines and perpetuating ECM breakdown.^11^ However, how pathological factors such as age, sex, hypertension, and smoking induce these changes in VSMCs remains poorly understood.

Recent studies have identified thioredoxin-interacting protein (TXNIP) as a critical mediator of oxidative stress and a multifunctional protein involved in various pathologies, including metabolic diseases and cancer.^12^^,^^13^^,^^14^ TXNIP expression is tightly regulated at multiple levels during stress responses, including transcriptional regulation by glucose and insulin^14^^,^^15^ and translational initiation via internal ribosome entry sites (IRESs) in cancer cells.^16^ TXNIP has been implicated in cardiovascular diseases, including AAA, through the TXNIP-NLRP3 inflammasome, which mediates macrophage-driven inflammation.^17^ However, the role of VSMC-derived TXNIP in AAA remains unexplored, and the impact of AAA risk factors on TXNIP expression in VSMCs is unknown.

The N6-methyladenosine (m^6^A) RNA modification, a reversible epitranscriptomic process, has emerged as a regulator of AngII-induced aortic diseases. Increased m^6^A levels have been associated with AAA progression.^18^ This modification, catalyzed by a complex of “writers” (e.g., METTL3, METTL14, and WTAP), is erased by “erasers” (e.g., FTO and ALKBH5) and interpreted by “readers” (e.g., YTHDF family proteins).^19^^,^^20^ M^6^A methylation influences mRNA metabolism, including stability, splicing, translation, and secondary structure. YTHDF1 and YTHDF3 enhance translation efficiency, whereas YTHDF2 promotes mRNA degradation.

During stress, cells employ cap-independent mechanisms to maintain selective protein synthesis, crucial for adaptation.^21^^,^^22^ While transcriptome-wide m^6^A mapping studies are abundant, specific roles of mRNA m^6^A methylation remain underexplored. Our study investigated TXNIP regulation during AAA formation, revealing a novel m^6^A-mediated mechanism in VSMCs. We found that TXNIP upregulation in AAA tissue correlates with increased m^6^A methylation. METTL3 modulates TXNIP expression at the post-transcriptional level, while YTHDF1 and YTHDF2 regulate its stability and translation. Silencing YTHDF2 stabilized *TXNIP* mRNA, whereas YTHDF1 facilitated translation in response to AngII-induced stress. We identified m^6^A methylation in the 3′ untranslated region (UTR) of TXNIP as crucial for its cap-independent translation. Finally, silencing TXNIP in VSMCs prevented AAA formation, highlighting its pivotal role in disease progression.

## Results

### TXNIP expression in VSMCs is upregulated in AngII-induced AAA in mice

The TXNIP pathway plays a critical role in mediating oxidative stress, which contributes to several pathologies, particularly vascular inflammation.^13^ *TXNIP* knockout prevents atherosclerosis by inhibiting oxidative stress in VSMCs.^23^ To examine the role of *TXNIP* in AAA formation, we generated smooth-muscle-specific *TXNIP* knockout mice on an *ApoE*-deficient background (*ApoE*^−/−^ *TXNIP*^*SM−/−*^) and evaluated the effects 28 days after AngII or saline infusion in both *ApoE*^−/−^ *TXNIP*^*flox/flox*^ and *ApoE*^−/−^ *TXNIP*^*SM−/−*^ mice. As shown in Figure S1A, AngII infusion but not saline infusion, resulted in significant enlargement of the aortic layer in *ApoE*^−/−^*TXNIP*^*flox/flox*^ mice. Histology analysis H&E staining, Picrosirius Red staining, and Verhoeff-van Gieson staining of mouse aorta showed extensive vascular remodeling in AngII-infused *ApoE*^−/−^ *TXNIP*^*flox/flox*^ mice when compared to the saline-infused *ApoE*^−/−^ *TXNIP*^*flox/flox*^ mice (Figures S1B–S1D).

**Figure 1:**
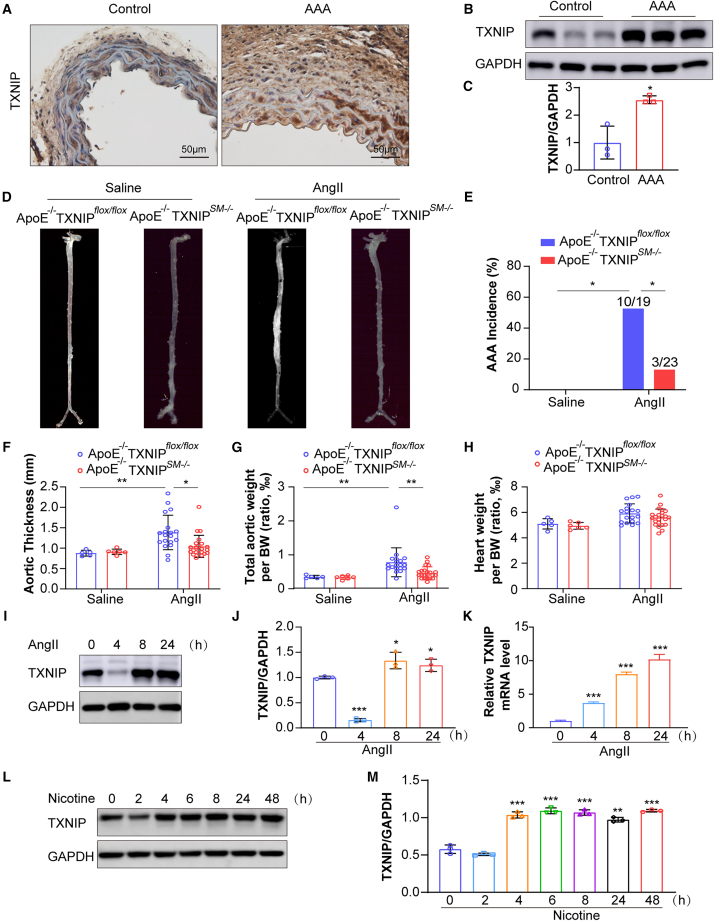
TXNIP expression is elevated in AngII-induced AAA in *ApoE*^−/−^ mice and in hASMCs treated with AngII or nicotine (A) Representative immunohistochemical staining of TXNIP in suprarenal aortas of *ApoE*^−/−^ mice infused with saline or AngII (1.44 mg/kg/day) using an osmotic mini-pump for 28 days (*n* = 5–22 mice/group). (B and C) Western blot analysis and quantification of TXNIP protein expression in suprarenal aortas from saline- or AngII-infused *ApoE*^−/−^ mice (*n* = 3 mice/group). (D) Representative images showing the macroscopic features of normal aorta and aneurysmal aorta induced by saline or AngII infusion for 28 days in the indicated group (*n* = 5–17 mice/group). (E) Incidence of AAA in each treatment group (*n* = 5–23 mice/group). (F–H) Quantification of (F) maximal aortic diameter, (G) total aortic weight, and (H) heart weight normalized to body weight (g) across the four mouse groups (*n* = 5–23 mice/group). (I and J) Western blot analysis and quantification of TXNIP protein expression in hASMCs after AngII treatment at different time points (*n* = 3 samples/group). (K) RT-qPCR quantification of *TXNIP* mRNA expression in hASMCs after AngII treatment at different time points (*n* = 3 samples/group). (L and M) Western blot analysis and quantification of TXNIP protein level in hASMCs treated with nicotine at different time points (*n* = 3 samples/group). Values are represented as mean ± SD; ∗*p <* 0.05; ∗∗*p <* 0.01; ∗∗∗*p <* 0.001 vs. control or 0 h.

Further, immunohistochemistry analysis revealed that TXNIP expression was markedly elevated in the medial layer of aortic tissue in AngII-infused *ApoE*^−/−^ *TXNIP*^*flox/flox*^ mice when compared to those in saline-treated *ApoE*^−/−^ *TXNIP*^*flox/flox*^ mice (Figure 1A). Western blot analysis of mouse aortas found a 2.5-fold increase in total TXNIP protein levels in AngII-infused *ApoE*^−/−^ *TXNIP*^*flox/flox*^ mice when compared to the saline-infused *ApoE*^−/−^ *TXNIP*^*flox/flox*^ mice (Figures 1B and 1C).

### Selective deletion of TXNIP in VSMCs in *ApoE*^−/−^ mice ablates AAA *in vivo*

As shown in Figures 1D and 1E, there was no AAA in either saline-infused *ApoE*^−/−^ *TXNIP*^*flox/flox*^ mice or saline-infused *ApoE*^−/−^ *TXNIP*^*SM−/−*^. In contrast, AngII infusion markedly increased AAA incidence in *ApoE*^−/−^ *TXNIP*^*flox/flox*^ mice (Figures 1D and 1E; Figure S1A). In AngII-infused *ApoE*^−/−^ *TXNIP*^*flox/flox*^ mice, AngII markedly resulted in increased aortic thickness (Figure 1F) and aortic weights (Figure 1G), while heart weight remained unchanged (Figure 1H). Morphologically, AngII infusion in *ApoE*^−/−^ *TXNIP*^*flox/flox*^ mice caused elastin degradation, elastic fiber fragmentation, and collagen deposition in the aortic wall (Figures S1B–S1D).

In contrast, AngII-infused *ApoE*^−/−^ *TXNIP*^*SM−/−*^ mice exhibited a phenotype similar to saline-treated controls (Figure 1D). Compared to AngII-infused *ApoE*^−/−^ *TXNIP*^*flox/flox*^ mice, the AAA incidence in *ApoE*^−/−^ *TXNIP*^*SM−/−*^ mice was significantly reduced to 15% (Figure 1E; Figure S1A). Both aortic thickness and weight were also significantly lower in *ApoE*^−/−^ *TXNIP*^*SM−/−*^ mice compared to their *ApoE*^−/−^ *TXNIP*^*flox/flox*^ counterparts (Figures 1F and 1G). Histological analyses showed elastin degradation, elastic fiber fragmentation, and collagen deposition in the aortic walls were less in AngII-infused *ApoE*^−/−^ *TXNIP*^*SM−/−*^ mice when compared to those in *ApoE*^−/−^ *TXNIP*^*flox/flox*^ (Figures S1B–S1D), indicating that VSMC-specific deletion of TXNIP lessened AngII-induced elastic fiber degradation or collagen accumulation (Figures S1B–S1D).

Matrix metalloproteinase (MMP) 2 activation plays key role in matrix remodeling of AAA.^4^^,^^24^ To further evaluate the impact of TXNIP on aortic remodeling in AAA, we assayed MMP2 expression and MMP2 activity in the mouse aorta images of suprarenal aortas of *ApoE*^−/−^ *TXNIP*^*flox/flox*^ mice and *ApoE*^−/−^ *TXNIP*^*SM−/−*^ mice infused with saline or AngII (1.44 mg/kg/day) using an osmotic mini-pump for 28 days. As shown in Figures S2A–S2C, AngII infusion caused higher expression and activity of MMP2 in *ApoE*^−/−^ *TXNIP*^*flox/flox*^ mice than their counterparts of *ApoE*^−/−^ *TXNIP*^*SM−/−*^ mice *in vivo*. Altogether, these results indicate that VSMC-specific *TXNIP* ablation protects against AngII-induced AAA development in *ApoE*^−/−^ mice.

### AngII or nicotine increase TXNIP via transcriptional and post-transcriptional manner in cultured VSMCs

To investigate TXNIP expression *in vitro*, human aortic vascular smooth muscle cells (hASMCs) were stimulated with AngII at various time points. Consistent with *in vivo* results, TXNIP protein levels were upregulated following AngII treatment (Figures 1I and 1J). RT-qPCR showed a robust increase in *TXNIP* mRNA levels (Figure 1K). Given the well-established role of tobacco as a risk factor for AAA,^4^ we also examined TXNIP expression in response to nicotine. TXNIP protein levels were significantly elevated after 4 h of nicotine exposure (Figures 1L and 1M). These findings suggest that TXNIP expression is regulated at both transcriptional and post-transcriptional levels during AngII-induced AAA.

### m^6^A methylation is increased in AAA and stress-induced VSMCs

**Figure 2:**
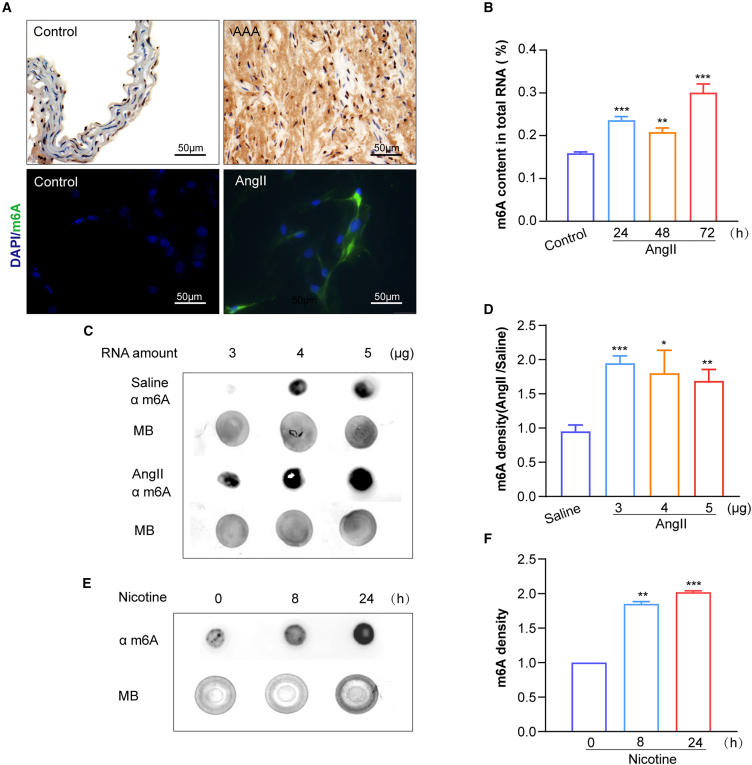
AngII enhances m^6^A modification in VSMCs *in vivo* and *in vitro* (A) Representative images of m^6^A obtained through immunostaining in the suprarenal aortas of *ApoE*^−/−^ mice infused with AngII (1.44 mg/kg/day) using an osmotic mini-pump for 28 days (*n* = 3 mice/group). Additionally, m^6^A immunofluorescence was performed on hASMCs treated with or without AngII (1 μM). (B) Quantification of m^6^A levels in total RNA from hASMCs treated with AngII (1 μM) for 24, 48, and 72 h (*n* = 5 samples/group). (C and D) m^6^A RNA immuno-dot blot and m^6^A quantification for saline and AngII (1 μM) treatment. Different amounts of RNA were loaded, and methylene blue was used as the loading control (*n* = 3 samples/group). (E and F) m^6^A RNA immuno-dot blot and m^6^A quantification after nicotine treatment (1.25 μM) at 0, 8, and 24 h, and methylene blue was used as the loading control (*n* = 5 samples/group). Values are represented as mean ± SD; ∗*p* < 0.05; ∗∗*p* < 0.01; ∗∗∗*p* < 0.001 vs. control, saline, or 0 h.

m^6^A methylation, an internal mRNA modification, modulates gene expression to adapt to environmental stimuli and is associated with various physiological and pathological processes in eukaryotes.^25^ To examine RNA modification changes in AAA pathology, we utilized the AngII-induced *ApoE*^−/−^ mouse model, a widely accepted system for studying AAA formation.^26^ Immunostaining analysis revealed a significant increase in nuclear m^6^A levels in AngII-treated AAA tissues and VSMCs (Figure 2A).

To validate these findings, we quantified total m^6^A RNA levels in hASMCs following AngII treatment using two complementary methods: ELISA and m^6^A dot-blot (Figures 2B–2F). ELISA analysis demonstrated a significant elevation in m^6^A-methylated RNA levels after 24 h of treatment, with further increases observed at 72 h (Figure 2B). Similarly, m^6^A dot-blot assays confirmed enhanced m^6^A modifications in hASMCs treated with AngII or nicotine at 24 h (Figures 2C–2F). These results suggest that m^6^A plays a critical role as a gene expression modulator, highlighting extensive m^6^A-mediated RNA modifications that likely contribute to the regulatory network driving AAA progression.

### METTL3 is upregulated in the nucleus in response to AngII or nicotine in cultured VSMCs

**Figure 3:**
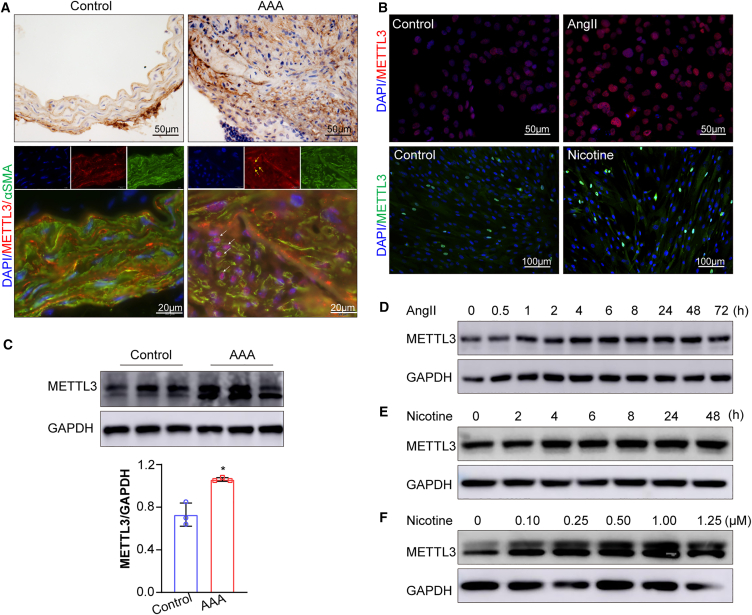
METTL3 is upregulated and distributed into the nucleus in VSMCs following AngII or nicotine stimulation *in vivo* and *in vitro* (A) Representative immunohistochemical and immunofluorescence images showing METTL3 expression and subcellular localization in aortic tissues from saline- or AngII-infused *ApoE*^−/−^ mice (*n* = 3 mice/group). (B) Representative immunofluorescence images of METTL3 localization in hASMCs treated with AngII or nicotine for 72 h (*n* = 3 samples/group). (C) Representative western blot analysis and quantification of METTL3 expression in the aortic tissues from saline- or AngII-infused *ApoE*^−/−^ mice (*n* = 3 mice/group). (D) Representative time course western blot analysis of METTL3 in hASMCs treated with AngII (*n* = 3 samples/group). (E) Representative time course western blot analysis of METTL3 in hASMCs treated with nicotine (*n* = 3 samples/group). (F) Representative dose-dependent western blot analysis of METTL3 in hASMCs treated with increasing concentrations of nicotine (*n* = 3 samples/group). Values are represented as mean ± SD; ∗*p* < 0.05 vs. control.

The m^6^A modification primarily depends on the catalytic activity of METTL3, a key methyltransferase localized in the nucleus.^27^ Given the observed increase in m^6^A RNA modifications following AngII treatment, we investigated whether METTL3 plays a role in AAA development. Immunostaining and immunofluorescence analysis revealed elevated METTL3 expression in the aortas of AngII-infused *ApoE*^−/−^ mice, with pronounced nuclear localization in AAA samples (Figures 3A and 3B). Western blot analysis of subcellular fractions confirmed that METTL3 is present in both nuclear and cytosolic compartments (Figure S3). Moreover, AngII stimulation in hASMCs markedly increased nuclear localization of METTL3 (Figure S3).

Protein extraction from aortic tissue confirmed increased METTL3 levels in AAA-affected aortas (Figure 3C). *In vitro*, METTL3 expression was upregulated in a time-dependent manner following AngII treatment (Figure 3D). Additionally, METTL3 expression showed significant time- and dose-dependent increases in response to nicotine (Figures 3E and 3F). These findings demonstrate that METTL3 expression is upregulated and that the protein is distributed in both the nucleus and cytosol in response to AngII or nicotine, suggesting its involvement in RNA methylation during AAA progression.

### METTL3 modulates TXNIP expression at the post-transcriptional level

**Figure 4:**
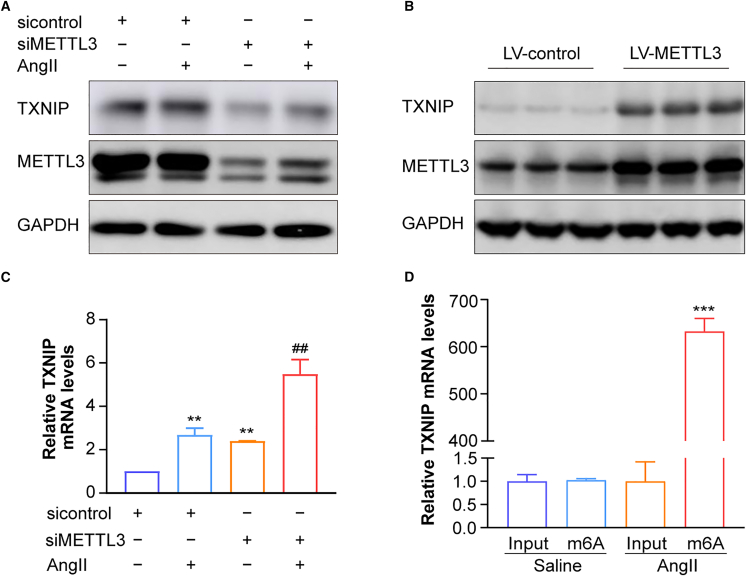
METTL3 modulates TXNIP expression at the post-transcriptional level via m^6^A modification in hASMCs under AngII stimulation (A) Representative western blots for TXNIP and METTL3 protein levels in hASMCs transfected with METTL3 siRNA (siMETTL3) or control siRNA (sicontrol) with or without AngII treatment (*n* = 3 samples/group). (B) Representative western blot analysis of TXNIP and METTL3 expression following METTL3 overexpression in hASMCs (*n* = 3 samples/group). (C) RT-qPCR analysis of *TXNIP* mRNA levels in METTL3-silenced hASMCs, with or without AngII treatment (*n* = 3 samples/group). (D) m6A-RIP-qPCR analysis of *TXNIP* mRNA methylation in hASMCs treated with or without AngII (1 μM) (*n* = 3 samples/group). Values are represented as mean ± SD; ∗∗*p* < 0.01; ∗∗∗*p* < 0.001 vs. sicontrol; ^##^*p* < 0.01 vs. siMETTL3.

METTL3 plays a crucial role in initiating m^6^A methylation on mRNA. To investigate its impact on TXNIP expression, we employed gain- and loss-of-function approaches targeting METTL3. In hASMCs, silencing METTL3 markedly reduced TXNIP protein expression (Figure 4A). Interestingly, METTL3 knockdown affected TXNIP expression even under basal conditions, independent of AngII stimulation. For the gain-of-function analysis, due to low transfection efficiency with plasmids, we subcloned METTL3 into lentiviral vectors. Overexpression of METTL3 in hASMCs significantly upregulated TXNIP protein levels (Figure 4B). These findings suggest that METTL3 regulates TXNIP expression.

Next, we examined *TXNIP* mRNA levels upon *METTL3* silencing (Figure 4C). Paradoxically, *METTL3* knockdown led to an increase in *TXNIP* mRNA levels despite a reduction in its the protein expression, and this upregulation of *TXNIP* mRNA was further enhanced under AngII stimulation. This indicates that METTL3 likely regulates TXNIP at the translational level, as METTL3 inhibition led to mRNA accumulation due to impaired translation. To further explore the methylation of *TXNIP* mRNA, we used the m^6^A-RIP-qPCR technique to assess the interaction between *TXNIP* mRNA and *METTL3* (Figure 4D). Under AngII stimulation, significant m^6^A modification of *TXNIP* mRNA was detected compared to controls (Figure 4D). These results suggest that TXNIP transcripts undergo m^6^A methylation, likely catalyzed by METTL3, highlighting its role in post-transcriptional regulation.

### Reduced YTHDF2 in AngII-induced AAA and AngII-treated VSMCs

**Figure 5:**
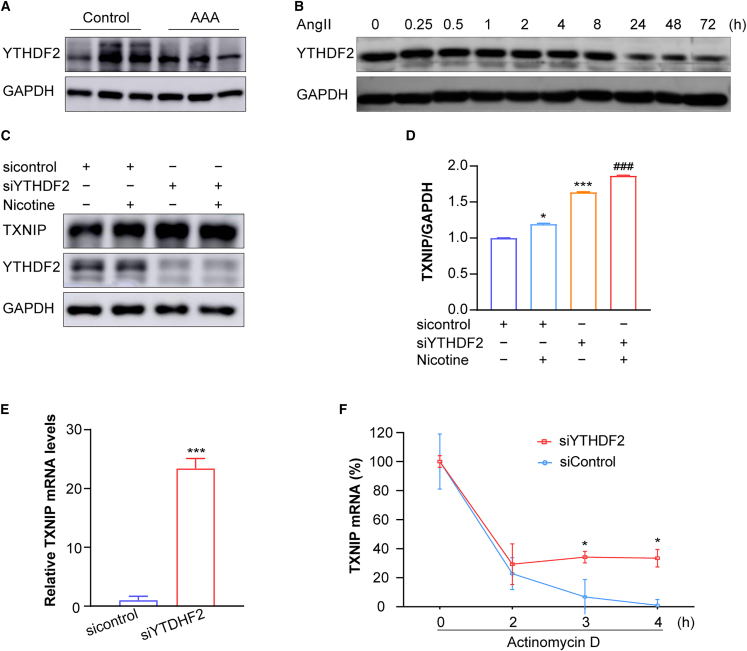
Silencing YTHDF2 enhances *TXNIP* mRNA stability in hASMCs (A) Representative western blot analysis of YTHDF2 level in saline- or AngII-infused *ApoE*^−/−^ mice (*n* = 3 mice/group). (B) Representative western blot analysis of YTHDF2 level in hASMCs treated with AngII (1 μM) at indicated time points (*n* = 3 samples/group). (C and D) Representative western blot analysis and quantification showing TXNIP expression in YTHDF2-silenced hASMCs following nicotine stimulation (*n* = 3 samples/group). (E) RT-qPCR analysis of *TXNIP* mRNA levels in YTHDF2-silenced hASMCs (*n* = 3 samples/group). (F) *TXNIP* mRNA stability analysis after actinomycin-D treatment in time-dependent manner (*n* = 3 samples/group). Values are represented as mean ± SD; ∗*p* < 0.05; ∗∗∗*p* < 0.001 vs. sicontrol; ^###^*p* < 0.001 vs. siYTHDF2.

M^6^A-modified mRNAs are recognized by specific proteins called “readers”, which mediate RNA processing. Among these, YTHDF2 plays a crucial role in regulating RNA stability, often promoting the degradation of its methylated target mRNAs.^19^ To examine the role of YTHDF2 in AAA, we first assessed its expression using western blot analysis. In AngII-induced AAA tissue, YTHDF2 expression was significantly suppressed (Figure 5A). This finding was consistent with *in vitro* results, where AngII treatment inhibited YTHDF2 expression in hASMCs (Figure 5B).

### Silencing YTHDF2 increases *TXNIP* mRNA stability

Functionally, silencing YTHDF2 increased TXNIP protein expression under basal conditions and further amplified TXNIP levels in response to nicotine treatment in hASMCs (Figures 5C and 5D). At the transcript level, YTHDF2 knockdown elevated *TXNIP* mRNA (Figure 5E). To explore the role of YTHDF2 in *TXNIP* mRNA stability, we silenced YTHDF2 and treated hASMCs with actinomycin D for 2, 3, and 4 h to inhibit transcription (Figure 5F). By 3 and 4 h, *TXNIP* mRNA was more stable in YTHDF2-silenced cells compared to small interfering RNA (siRNA) controls. Altogether, these findings demonstrate that YTHDF2 expression is inversely correlated with TXNIP levels under AngII-induced stress. Suppression of YTHDF2 stabilizes *TXNIP* mRNA, leading to its increased expression. Thus, in response to AngII-induced stress, the reduction in YTHDF2 levels enhances *TXNIP* mRNA stability, promoting its upregulation.

### TXNIP translation is cap-independent

**Figure 6:**
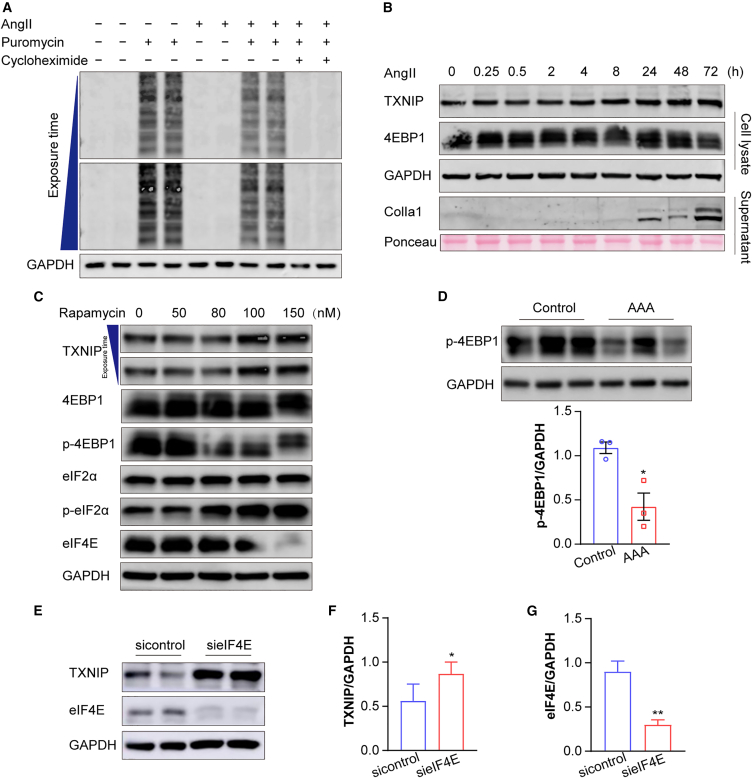
TXNIP is upregulated by AngII in a cap-independent manner in VSMCs (A) Representative imagines for puromycin incorporation assay performed in primary VSMCs isolated from *ApoE*^−/−^ mice to assess global protein synthesis (*n* = 3 samples/group). (B) Representative western blots showing TXNIP and 4EBP1 expression in whole-cell lysates and Col1a1 secretion in the culture supernatants of hASMCs following AngII (1 μM) treatment at various time points. GAPDH and Ponceau S staining were used as loading controls (*n* = 3 samples/group). (C) Representative western blot analysis of TXNIP and key regulators of translational control, including 4EBP1, phosphorylated 4EBP1 (p-4EBP1), eIF4E, eIF2α, and phosphorylated eIF2α (p-eIF2α), following rapamycin treatment at different concentrations in hASMCs (*n* = 3 samples/group). (D) Western blots analysis and quantification of p-4EBP1 levels in the suprarenal aortas of saline- or AngII-infused *ApoE*^−/−^ mice (*n* = 3 mice/group). (E–G) Representative western blots and quantification showing TXNIP and eIF4E expression in hASMCs following eIF4E knockdown (*n* = 3 samples/group). Values are represented as mean ± SD; ∗*p* < 0.05 vs. control or sicontrol; ∗∗*p* < 0.01 vs. sicontrol.

M^6^A methylation is a well-established regulator of gene expression, particularly by promoting translation.^28^ Under cellular stress, such as that induced by pathological or physiological conditions, global cap-dependent protein synthesis is often inhibited. However, certain mRNAs can bypass this inhibition through cap-independent translation mechanisms. Notably, m^6^A modifications can facilitate cap-independent translation during pathological conditions.^29^ To investigate this, we first analyzed global protein synthesis in AngII-treated hASMCs using a puromycin assay (Figure 6A). Puromycin incorporation into nascent peptides was reduced following AngII stimulation, indicating a decrease in global translation. Cycloheximide served as a control to confirm this inhibition. Next, we examined TXNIP protein expression at various time points after AngII treatment (Figure 6B). TXNIP was actively translated between 24 and 72 h, a time frame that coincided with increased collagen ColIa1 protein expression.

Rapamycin, a potent mTORC1 inhibitor, suppresses cap-dependent translation initiation by promoting 4E-BP1 hypophosphorylation, thereby enhancing its association with eIF4E.^30^^,^^31^ Notably, as shown in Figure 6C, high-dose rapamycin treatment (100–150 nM) led to a robust increase in TXNIP protein levels, despite simultaneous induction of 4E-BP1 hypophosphorylation and eIF2α hyperphosphorylation—both well-established indicators of translational repression. This discrepancy suggests that the elevation of TXNIP is unlikely to result from enhanced cap-dependent translation.

Consistently, *in vivo* analyses revealed that phosphorylated 4EBP1 levels were reduced in AngII-infused AAA tissues but remained high in vehicle-treated controls (Figure 6D). Finally, to directly test the cap-independent translation of TXNIP, we silenced eIF4E in hASMCs. Interestingly, TXNIP expression was significantly elevated in the absence of eIF4E (Figures 6E–6G), supporting the hypothesis that TXNIP translation is cap-independent.

Taken together, these data suggest that AngII-induced stress activates TXNIP translation through a cap-independent mechanism in VSMCs.

### *TXNIP* mRNA translation is dependent on YTHDF1 binding

**Figure 7:**
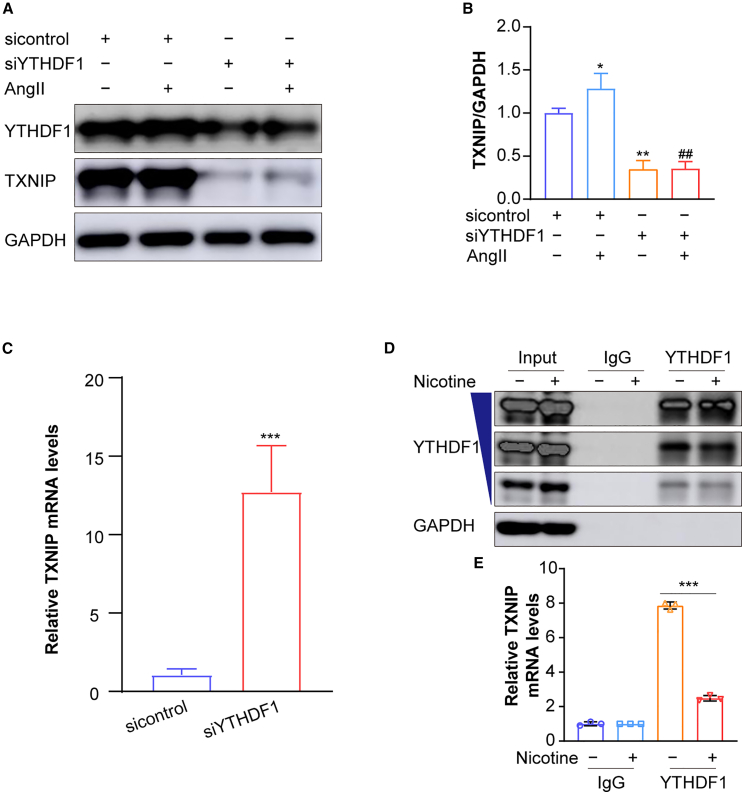
TXNIP translation is dependent on the m^6^A reader YTHDF1 in hASMCs (A and B) Representative western blots and quantification of TXNIP expression in YTHDF1-silenced hASMCs following AngII (1 μM) treatment (*n* = 3 samples/group). (C) RT-qPCR analysis of *TXNIP* mRNA in YTHDF1-silenced hASMCs (*n* = 3 samples/group). (D) Representative western blot showing the successful immunoprecipitation of YTHDF1 protein in RIP samples using an anti-YTHDF1 antibody. Immunoglobulin G (IgG) served as the negative control (*n* = 3 samples/group). (E) RT-qPCR analysis of *TXNIP* mRNA enrichment in RIP assays using an anti-YTHDF1 antibody (*n* = 3 samples/group). Values are represented as mean ± SD; ∗*p* < 0.05; ∗∗*p* < 0.01; ∗∗∗*p* < 0.001 vs. sicontrol; ^##^*p* < 0.01 vs. siYTHDF1.

The translation of m^6^A-modified mRNA often relies on m^6^A reader proteins, with YTHDF1 serving as a key enhancer of translation.^32^ YTHDF1 has been shown to facilitate ribosome loading to activate translation.^33^ To investigate YTHDF1’s role in regulating TXNIP expression, we silenced YTHDF1 and examined TXNIP levels. Silencing YTHDF1 significantly reduced TXNIP protein expression (Figures 7A and 7B). However, *TXNIP* mRNA levels increased under the same conditions (Figure 7C), likely due to the accumulation of untranslated mRNA when translation is inhibited. These findings indicate that YTHDF1 plays a critical role in the translation of TXNIP.

As an m^6^A reader protein, YTHDF1 is known to bind directly to m^6^A-modified mRNA. To determine whether YTHDF1 interacts with TXNIP transcripts, we evaluated their binding under both normal and nicotine-stimulated conditions. We found that YTHDF1 binds to *TXNIP* mRNA under basal conditions (Figure 7D). Although this interaction was maintained in nicotine-treated cells, its strength was markedly reduced compared to normal conditions (Figure 7E). These results suggest that YTHDF1 recognizes methylated *TXNIP* mRNA and modulates its translation, thereby regulating TXNIP protein production.

### TXNIP methylation occurs in 3′UTR

**Figure 8:**
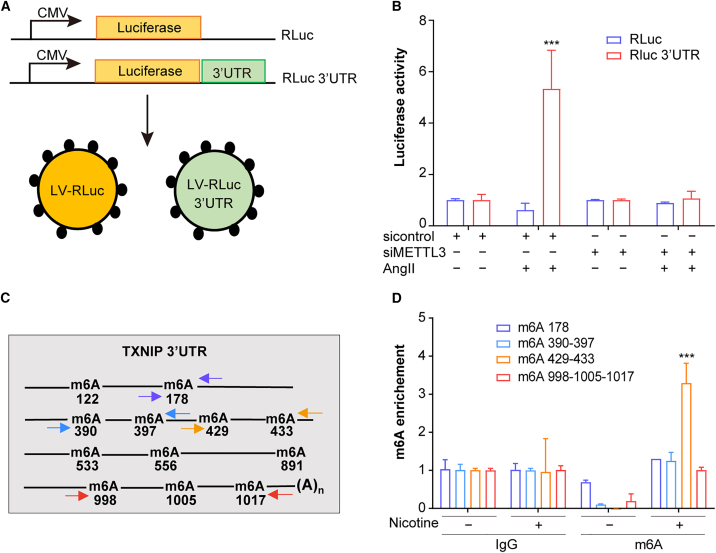
TXNIP methylation occurs in 3′UTR in hASMCs (A) Schematic representation of the construction of reporter plasmids and generation of the corresponding lentiviral vectors. (B) Luciferase activity in METTL3-silenced and control hASMCs, with or without AngII (1 μM) treatment (*n* = 3 samples/group). (C) Schematic of the 3′UTR of *TXNIP* mRNA indicating predicted m^6^A sites based on multiple m^6^A-sequencing databases. (D) MeRIP followed by RT-qPCR using different primer sets targeting the 3′UTR of *TXNIP*, as illustrated in (C) (*n* = 3 samples/group). Values are represented as mean ± SD; ∗∗∗*p* < 0.001.

In most cases, UTRs in the 3′ or 5′ ends play a crucial role in translational control by interacting with RNA-binding proteins. Translation initiation is facilitated when YTHDF1 binds to the 3′UTR of methylated mRNA.^33^ Meyer et al.’s analysis revealed a significant enrichment of m^6^A methylation in the 3′UTR of mRNAs.^34^^,^^35^ To investigate the role of the 3′UTR in m^6^A-dependent translation of TXNIP, we utilized a luciferase reporter assay. Two vectors were constructed: one containing luciferase alone as a control (RLuc) and another with luciferase followed by the 3′ UTR sequence of TXNIP (RLuc-3′UTR) (Figure 8A). In stress-induced cells, luciferase activity was significantly higher for the RLuc-3′UTR compared to the control RLuc (Figure 8B). Notably, upon METTL3 knockdown, luciferase activity from the RLuc-3′UTR construct decreased to levels comparable to the control RLuc (Figure 8B). These results suggest that methylation within the 3′UTR of *TXNIP* mRNA promotes its translational activation.

SRAMP analysis identified 12 m^6^A sites in the 3′UTR of *TXNIP* (Figure 8C). To pinpoint the specific methylation sites, we performed m^6^A-RIP followed by RT-qPCR using primers targeting the predicted m^6^A sites. The results showed enrichment of m^6^A at positions 429 and 433 in stress-induced cells. These findings indicate that, in response to stress, *TXNIP* mRNA undergoes methylation at adenosine positions 429 and 433 within its 3′UTR, triggering translation initiation. Additionally, m^6^A-RIP and RT-qPCR in nicotine-treated cells revealed a significant increase in methylation at the 429-433 adenosine positions within the 3′UTR of *TXNIP* mRNA (Figure 8D). These results further support the role of m^6^A methylation in regulating TXNIP translation under stress conditions.

## Discussion

This study uncovers a critical role for TXNIP in AAA development and provides mechanistic insights into its regulation via m^6^A RNA methylation and translational control. Our results show that stress-induced conditions, such as AngII and nicotine exposure, significantly upregulate TXNIP expression in VSMCs at transcriptional and post-transcriptional levels. *In vivo*, TXNIP expression is associated with enhanced AAA formation in *ApoE*^−/−^ mice, while smooth-muscle-specific deletion of TXNIP protects against AngII-induced AAA development. Additionally, we demonstrate that m^6^A RNA methylation, driven by METTL3, plays a pivotal role in regulating TXNIP translation through interactions with YTHDF1, a key m^6^A reader protein. Furthermore, *TXNIP* mRNA undergoes m^6^A modifications at specific sites within its 3′UTR, enabling cap-independent translation, particularly under stress conditions that inhibit global cap-dependent translation.

VSMCs play a critical role in AAA pathogenesis, as they contribute to extracellular matrix degradation, which promotes aneurysm formation.^10^ Our findings reveal for the first time that smooth-muscle-specific deletion of TXNIP protects against AngII-induced AAA in *ApoE*^−/−^ mice. TXNIP exerts pleiotropic effects: it induces apoptosis in β-cells^36^ while acting as a tumor suppressor in breast cancer.^37^ In atherosclerosis, global *TXNIP* knockout in *ApoE*^−/−^ mice reduces inflammation in VSMCs.^38^ In the context of AAA, *TXNIP* deletion in VSMCs appears to exert anti-apoptotic and anti-inflammatory effects, preserving cellular integrity and inhibiting aneurysm expansion. Our results, combined with prior findings, suggest that cell-type specificity is crucial to the pathological role of TXNIP.

In this study, we provide the first evidence that m^6^A-dependent control of TXNIP translation initiation occurs in VSMCs, contributing to the progression of AAA. Using VSMCs, we identified enriched m^6^A sites at positions 429–433 in the 3′UTR of TXNIP mRNA. We also identified the involvement of the YTH domain family proteins YTHDF1 and YTHDF2 in the post-transcriptional regulation of TXNIP. While YTHDF2 primarily mediates mRNA stability,^19^ YTHDF1 promotes translational efficiency.^39^^,^^40^ In AAA pathogenesis and cultured VSMCs, YTHDF2 levels were significantly reduced, leading to increased stability of *TXNIP* mRNA, while YTHDF1 enhanced its translation. This suggests that YTHDF1 and YTHDF2 coordinate TXNIP regulation in response to stress. Notably, *TXNIP* mRNA bound to YTHDF1 even under normal conditions, suggesting an additional cap-dependent regulatory mechanism.

The regulation of TXNIP has been extensively studied, particularly in response to glucose. TXNIP expression is strongly induced at the transcriptional level by glucose.^14^ Conversely, TXNIP is sensitive to insulin and transcriptionally regulated by the transcription factor FOXO1.^15^^,^^41^ At the translational level, TXNIP regulation has been associated with ribosomal elongation^42^ and IRES-mediated initiation in cancer cells.^16^ Post-translationally, TXNIP is phosphorylated by AKT and AMPK in response to insulin and glucose, respectively.^43^^,^^44^ The m^6^A methyltransferase METTL3 also plays a key role in TXNIP regulation. While METTL3 is catalytically active in the nucleus, it has been shown to interact with the cytosolic translation initiation complex during cap-dependent translation.^45^ In our study, stress-induced nuclear translocation of METTL3 enhanced m^6^A methylation of *TXNIP* mRNA, thereby promoting its translation. Silencing METTL3 reduced *TXNIP* mRNA levels, suggesting a potential role for METTL3 in mRNA stability and/or transcription that warrants further exploration.

Epitranscriptomics represents a critical layer of post-transcriptional regulation in RNA metabolism, including splicing, export, and translation.^46^ Recent studies have highlighted the significant role of m^6^A modifications in physiological and pathological processes, including cardiovascular diseases.^47^^,^^48^^,^^49^ Despite the widespread mapping of m^6^A sites across mRNA transcripts, functional characterization of specific m^6^A targets remains limited. This study is the first to demonstrate that m^6^A methylation of TXNIP plays a key role in its gene regulation. Our findings indicate that m^6^A methylation within the 3′UTR of *TXNIP* mRNA promotes its translation, especially after 24 h, underscoring the importance of untranslated regions in mRNA stability and translation. Interactions between the 3′UTR, 5′UTR, and poly(A)-binding protein (PABP) may form regulatory loops critical for TXNIP expression, which merits additional investigation. Collectively, this study proposes a novel regulatory mechanism whereby m^6^A-dependent translation of TXNIP contributes to AAA development. Targeting m^6^A methylation of *TXNIP* mRNA offers a promising therapeutic strategy for AAA and potentially other pathologies.

The broader implications of our findings might well extend beyond AAA, as the TXNIP-m^6^A axis may play a role in other stress-induced vascular diseases, such as atherosclerosis and hypertension.^50^^,^^51^ These insights open new avenues for therapeutic intervention in AAA and related vascular conditions, including atherosclerosis and cardiac hypertrophy, that involve oxidative and epitranscriptional stress. By demonstrating the involvement of m^6^A RNA methylation in VSMC dysfunction, this study highlights the potential of targeting epitranscriptional modifications as a therapeutic strategy. Specifically, inhibiting METTL3 or modulating YTHDF1 activity could offer novel approaches to mitigate pathological TXNIP upregulation without broadly suppressing TXNIP’s physiological functions.

Moreover, our data on cap-independent translation suggests a paradigm shift in understanding gene expression during stress. The ability of TXNIP to bypass global translational inhibition may represent a generalizable mechanism for other stress-responsive genes, emphasizing the need for further research into the role of 3′ UTRs and m^6^A modifications in translational control. These findings also underscore the importance of environmental factors, such as smoking, in vascular pathology, reinforcing public health initiatives aimed at reducing tobacco use.

### Limitations of the study

Despite these advances, several limitations should be acknowledged. First, although the *in vivo* studies using ApoE^−/−^ TXNIP^SM−/−^ mice provide compelling evidence for TXNIP’s role in AAA, additional models—particularly humanized systems—would further strengthen the translational relevance of the findings. Second, the regulatory pathways linking m^6^A methylation, YTHDF1, and TXNIP remain incompletely defined. Further investigation is needed to elucidate their interactions under varying stress conditions. In this regard, MeRIP-seq analysis in hASMCs would offer a more precise method to confirm TXNIP mRNA methylation within the 3′UTR and to pinpoint specific modification sites. Third, although our data support cap-independent translation of TXNIP, the mechanisms governing ribosome recruitment to the 3′UTR are still speculative and require additional study. Moreover, the basis for the selective enrichment of m^6^A modifications within the TXNIP 3′UTR remains unclear and warrants further investigation. The use of METTL3 inhibitors in both *in vitro* and *in vivo* models could help validate these observations and assess their potential therapeutic value in preventing AAA. Finally, given that nicotine and AngII represent distinct stress stimuli, comparative studies examining their differential effects on TXNIP regulation would provide a more comprehensive understanding across diverse pathological contexts.

In conclusion, our study provides compelling evidence that TXNIP is a critical mediator of AAA progression, regulated by m^6^A RNA methylation and translational control. These findings advance our understanding of the molecular mechanisms underlying AAA progression and highlight the TXNIP-m^6^A axis as a potential therapeutic target. Specifically, the upregulation of TXNIP in VSMCs links oxidative stress and vascular remodeling to AAA pathology, corroborating previous studies on the role of TXNIP in atherosclerosis. Importantly, the identification of METTL3-mediated m^6^A methylation and YTHDF1-dependent translation as key regulatory mechanisms offers a novel perspective on how TXNIP expression is controlled during stress.

## Resource availability

### Lead contact

Further information and requests for resources and reagents should be directed to the lead contact, Ming-Hui Zou (mhzou@tmu.edu.cn).

### Materials availability

This study did not generate new unique reagents.

### Data and code availability

• All data in this paper will be shared by the lead contact upon request.
• This study did not generate original code.
• Any additional information required to reanalyze the data reported in this paper is available from the lead contact upon request.

## Acknowledgments

We greatly appreciated the technical assistance and helpful discussion from Drs. Tatiana Bedarida and Qiulun LV. This work was supported in part by funding from the Nature Science Foundation of China (82470443 and 82530049) and the Transformational Award of 10.13039/100000968American Heart Association, USA.

## Author contributions

M.H.Z. conceived the project. M.H.Z. designed the research. F.H. carried out the experiments and collected the data. F.H. and W.Y. analyzed the data and prepared the figures. F.H. and W.Y. wrote the article. M.H.Z. revised the manuscript, supervised the research, and provided the funding. All authors approved the submitted and published version.

## Declaration of interests

The authors declare no conflict of interest.

## STAR★Methods

### Key resources table

**Table:** 

REAGENT or RESOURCE	SOURCE	IDENTIFIER
**Antibodies**		

anti-METTL3	Proteintech	Cat#15073-1-AP
anti-METTL3	Abcam	Cat#ab195352
anti-TXNIP	Cell Signaling Technology	Cat#14715S
anti-m6A	Abcam	Cat#ab151230
anti-m6A	Synaptic Systems	Cat#202003
anti-YTHDF1	Proteintech	Cat#17479-1-AP
anti-Phospho-4E-BP1 (Thr37/46)	New England Biolabs	Cat#R3133S
anti-YTHDF2	Proteintech	Cat#24744-1-AP
anti-puromycin	Kerafast	Cat#EQ0001
anti-Colla1	Abcam	Cat#ab7046
anti-4EBP1	Abcam	Cat#ab278688

**Chemicals, peptides, and recombinant proteins**		

Angiotensin II human	Millipore Sigma	Cat#A9525
Actinomycin D	Thermo Fischer Scientific	Cat#11805017
Rapamycin	Millipore Sigma	Cat#R0395

**Critical commercial assays**		

Renilla-Glo Luciferase Assay System	Promega	Cat#E2710
Lipofectamine RNAiMax	Thermo Fischer Scientific	Cat#13778150
GenElute™ mRNA Miniprep Kit	Sigma-Aldrich	Cat#MRN10
Magna MeRIP m^6^A Kit	Millipore	Cat#17-10499
Zero Blunt TOPO PCR Kit	Thermo Fischer Scientific	Cat#K280002

**Experimental models: Cell lines**		

Human: hASMCs	Clonetics	Cat#CC-2571

**Experimental models: Organisms/strains**		

Mouse: WT, C57BL/6	The Jackson Laboratory	N/A
Mouse: *ApoE*^−/−^	The Jackson Laboratory	N/A
Mouse: *TXNIP*^*flox/flox*^	The Jackson Laboratory	N/A

**Oligonucleotides**		

eIF4E siRNA	Santa Cruz	Cat#SC-35284
ON-TARGETplus Human YTHDF1 siRNA	Horizon	Cat#54915
ON-TARGETplus Human YTHDF2 siRNA	Horizon	Cat#51441
ON-TARGETplus Human METTL3 siRNA	Horizon	Cat#56339

**Recombinant DNA**		

pcDNA3/Flag-METTL3	Addgene	Plasmid #53739
plenti-CMV MCS-GFP-SV-puro	Addgene	Plasmid #73582

**Software and algorithms**		

GraphPad Prism 4	GraphPad Software, LLC	https://www.graphpad.com/
ImageJ	ImageJ	https://imagej.net/ij/

### Experimental model and study participant details

#### Animals

Male wild-type (WT, C57BL/6) and *ApoE*^−/−^ mice (12–16 weeks old; 20–25 g body weight) were obtained from Jackson Laboratories (Bar Harbor, ME). Vascular smooth muscle cell-specific TXNIP knockout mice (*TXNIP*^*SM−/−*^) were generated as described previously.^38^ *TXNIP*^*SM−/−*^ mice on a C57BL/6 background were crossed with *ApoE*^−/−^ mice to generate *ApoE*^−/−^ *TXNIP*^*SM−/−*^ mice. *ApoE*^−/−^ *TXNIP*^*flox/flox*^ mice served as controls. All mice were housed at Georgia State University animal facility in individually ventilated cages in temperature-controlled cages under a 12-h light-dark cycle with free access to food and water. Adult male mice were randomly assigned to experimental groups and female mice were excluded from the study, as male mice reliably and robustly develop aneurysms under AngII infusion, mirroring human sex difference in AAA prevalence. Animal protocol (A22001)was reviewed and approved by the Georgia State University Institutional Animal Care and Use Committee.

#### Cell culture and treatments

##### Primary cells

Mouse VSMCs were isolated from aortas of *ApoE*^−/−^ *TXNIP*^*flox/flox*^ or *ApoE*^−/−^ *TXNIP*^*SM−/−*^ mice. Aortas were washed, cleaned of fat and connective tissue, and cut into 3-mm sections. After endothelial removal, tissues were digested in 0.2% collagenase at 37°C. Detached VSMCs were centrifuged, washed, and plated in M231 medium. Cell purity was confirmed by α-SMA staining, and passages 3–5 were used.

Human aortic vascular smooth muscle cells (hASMCs, Clonetics) were cultured in M231 medium supplemented with 10% FBS, penicillin (100 U/mL), and streptomycin (100 μg/mL) at 37°C in a humidified 5% CO_2_ atmosphere. At 70–80% confluency, hASMCs grown in M231 medium were treated with different agents, as indicated. The purity of cells was confirmed through positive staining for α-SMA. In all experiments, cells were used between passages 3 and 10. For experiments involving AngII or nicotine treatment, cells were treated with AngII or nicotine at the concentrations indicated for 24 h unless otherwise stated.

### Method details

#### Analysis and quantification of AAA

Mice (*ApoE*^−/−^ *TXNIP*^*flox/flox*^, *ApoE*^−/−^ *TXNIP*^*SM−/−*^) fed a normal chow diet were infused with AngII (1.44 mg/kg/day for 4 weeks) using Alzet osmotic pumps (DURECT Corp). Mice were anesthetized via intraperitoneal injection of ketamine (80 mg/kg) and xylazine (5 mg/kg). Pumps were implanted subcutaneously through a small incision at the nape, which was closed with sutures. Incision sites healed without infection. AAA incidence and size were quantified by measuring the maximum width of the abdominal aorta using Image Pro Plus software (Media Cybernetics). Aneurysms were defined as an external suprarenal aortic width exceeding 50% of saline-infused controls (≥1.22 mm). This threshold aligns with clinical diagnostic standards for AAA.

#### Histological analysis

Following hemodynamic measurements, animals were sacrificed, and aortas were perfused with normal saline and fixed in 10% formalin at physiological pressure for 5 min. Whole aortas were harvested, fixed for 24 h, embedded in paraffin, and sectioned (5 μm). Sections were stained with Hematoxylin and Eosin or used for immunohistochemistry.

#### Immunohistochemistry and immunofluorescence

Formaldehyde-fixed paraffin sections were deparaffinized, rehydrated, and subjected to antigen retrieval before an overnight incubation at 4°C with primary antibodies for TXNIP, m6A, αSMA, or METTL3. Species- and isotype-matched IgG were used as negative controls. Slides were analyzed using an Olympus BX41 microscope with a Spot Insight 2 digital camera (Diagnostic Instruments). To account for lesion variability, three sections of the suprarenal aorta spaced 500 μm apart were analyzed, and vessel areas were measured using Image Pro Plus software.

#### Transfection of siRNA hASMCs

Transient transfection of siRNA was carried out according to manufacturer’s instruction. Briefly, the siRNAs were dissolved in siRNA buffer (20 mM KCl; 6 mM HEPES, pH 7.5; 0.2 mM MgCl_2_) to prepare a 10 μM stock solution. HASMCs grown in 6 well plates were transfected with siRNA in transfection medium containing liposomal transfection reagent (Lipofectamine RNAimax). For each transfection, 100 μL of transfection medium containing 4 μL siRNA stock solution was gently mixed with 100 μL transfection medium containing 4 μL transfection reagent. After a 30 min incubation at room temperature, siRNA-lipid complexes were added to the cells in 1.0 mL transfection medium, and cells were incubated with this mixture for 6 h at 37°C. The transfection medium was then replaced with normal medium, and cells were cultured for 48 h.

#### Western blot analysis

Cells were homogenized on ice in a cell lysis buffer containing 20 mM Tris-HCl (pH 7.5), 150 mM NaCl, 1 mM Na_2_EDTA, 1 mM EGTA, 1% Triton, 2.5 mM sodium pyrophosphate, 1 mM β-glycerophosphate, 1 mM Na_3_VO_4_, 1 μg/mL leupeptin, and 1 mM PMSF. Proteins were extracted by lysing cells with the lysis buffer, and their concentrations were determined using the BCA protein assay reagent (Pierce, USA). A total of 20 μg of protein was separated by SDS-PAGE and subsequently transferred onto a membrane. The membrane was incubated with a primary antibody, followed by a secondary antibody conjugated to horseradish peroxidase at a dilution of 1:2000. Protein bands were visualized using enhanced chemiluminescence (ECL, GE Healthcare). The intensity of individual bands was quantified via densitometry using FIJI software, with the background signal subtracted from the measured area. Results were normalized to the control, and/or non-treated which was set as 100%. Each Western blot is a representative of at least three blots from three independent experiments.

#### RNA extraction and RT-qPCR

Total RNA was extracted from tissues or cells using TRIzol (Ambion) according to the manufacturer’s instructions and quantified by UV spectrophotometry. Reverse transcription was conducted using PrimeScript RT Reagent Kit with gDNA Eraser (Takara, RR047B). RT-qPCR was then performed in triplicate on Light Cycler 480 II (Roche) using ChamQ Universal SYBR qPCR Master Mix (Vazyme, Q711-03). 18S was used as the internal control.

#### RNA m^6^A dot blot assays

The assay was used to determine the samples’ m^6^A concentrations. After the total RNA extraction from the aorta tissues and VSMCs, mRNA samples were denatured for 3 min at 95 °C and then quickly cooled on ice. UV light was used to crosslink it after adding a 2 μL sample drop to a nitrocellulose membrane (Amersham, USA). The membrane was blocked for 1 h at room temperature using 5% skimmed milk, and this was followed overnight at 4 °C with the m^6^A antibody. The next day, the membrane was incubated for 2 h at room temperature with HRP-coupled secondary antibodies before using ECL chemiluminescence for detection. Imaging was eventually performed using Image Lab (Bio-Rad).

#### m^6^A RNA immunoprecipitation assay (MeRIP)

As described in previous reports,^52^ mRNA m^6^A levels were determined with the Magna MeRIP m^6^A kit (Millipore, 17–10499) according to available instructions. The extracted RNA was fragmented into approximately 100 nucleotide long fragments in an RNA Fragmentation Buffer. The fragments were then purified and incubated with anti-m^6^A or control IgG for immunoprecipitation (IP). Finally, qPCR was performed on the IP production.

#### m^6^A-RIP-qPCR analysis of TXNIP m^6^A mRNA

The m^6^A-RIP-qPCR assay was performed as described previously.^53^ In summary, total RNA was extracted from hASMC, followed by the isolation of poly(A) mRNA using the GenElute mRNA Miniprep Kit (Sigma-Aldrich, MRN10). To minimize rRNA contamination, two rounds of purification were conducted according to the manufacturer’s instructions. Subsequently, 5 μg of mRNA was fragmented into 200–300 nucleotide segments by incubation in fragmentation buffer (10 mM ZnCl_2_, 10 mM Tris-HCl, pH 7.0) at 94 °C for 30 s. The reaction was halted with 50 mM EDTA, and the RNA fragments were purified via ethanol precipitation. The fragmented mRNA (5 μg) was incubated with 2 μg of anti-m^6^A antibody (Synaptic Systems, 202003) or mouse IgG (Beyotime, A7028) in 600 μL of RIP buffer (150 mM NaCl, 0.1% Igepal CA-630, 10 mM Tris-HCl, pH 7.4) at 4 °C for 2 h. Dynabeads Protein A (Thermo Fischer Scientific, 100-02D) and Protein G beads (Thermo Fischer Scientific, 100-04D) were then added, and the mixture was incubated at 4 °C for another 2 h. Beads were washed five times with RIP buffer, ensuring the presence of RNase inhibitor (Promega, N2611) throughout the process. Elution of mRNA fragments was achieved using 100 μL of 0.3 μg/μL Proteinase K (Thermo Fischer Scientific, AM2546) at 55 °C for 1 h, followed by phenol-chloroform extraction and ethanol precipitation. The resulting RNA was reverse transcribed, and the enrichment of m^6^A-modified fragments was analyzed via RT-qPCR.

#### RNA immunoprecipitation (RIP)-qPCR

The RIP-qPCR assay was performed following established protocols with slight modifications.^53^ HASMCs cultured in 15 cm dishes were lysed in 600 μL of homogenization buffer (100 mM KCl, 5 mM MgCl_2_, 10 mM HEPES, pH 7.0, 0.5% Nonidet P-40, 1 mM DTT, and 100 U/mL RNase inhibitor [Promega, N2611]). The homogenate was incubated on ice for 5 min and then centrifuged to collect the supernatant. Dynabeads Protein A (Thermo Fischer Scientific, 100-02D) and Protein G (Thermo Fischer Scientific, 100-04D) were incubated with 2 μg of YTHDF1 antibody (Proteintech, 17479-1-AP) at 4 °C for 2 h with gentle rotation. The antibody-bead complex was subsequently incubated with the homogenized supernatant under the same conditions. Beads were washed five times with washing buffer (50 mM Tris-HCl, pH 7.4, 150 mM NaCl, 1 mM MgCl_2_, 0.05% NP-40), and RNA was extracted using Trizol reagent. The extracted RNA was analyzed via RT-qPCR for downstream applications.

#### Plasmids construction and corresponding lentivectors production

The human METTL3 sequence was amplified from the plasmid pcDNA3/Flag-METTL3 and subsequently inserted into pCR-Topo-BluntII, resulting in the construction of pCR-Topo-BluntII-METTL3. The METTL3 cassette was digested with XbaI and MluI enzymes. The backbone of pLenti-CMV MCS-GFP-SV-puro was also digested with XbaI and MluI, removing the GFP sequence while retaining the backbone. The METTL3 sequence was then inserted into this backbone, resulting pLenti-CMV-METTL3. For the plasmid with reporter gene, pLenti-RLuc-3utrTXNIP was generated by separately subcloning RLuc and the 3utrTXNIP (2026 nt) sequence into pCR-Topo-BluntII. The RLuc cassette was digested using XbaI and PvuI, while the 3utrTXNIP was digested with PvuI and MluI. These cassettes were subsequently inserted into pLenti-CMV to produce pLenti-CMV-RLuc-3utrTXNIP. For control purposes, the RLuc cassette was digested with XbaI and MluI and inserted into pLenti-CMV-MCS. Lentivector particles were produced following the methodology described by Hantelys et al., in eLife, 2019.^54^

#### Luciferase activity assay

Luciferase activities were measured using a *Renilla*-Glo Luciferase Assay System (Promega, E2710) by following the manufacturer’s instructions. Briefly, proteins from cells were extracted, and the bioluminescence was quantified with a luminometer (Tecan).

#### mRNA stability assay

mRNA stability assay was performed in the presence of actinomycin D in cultured hASMCs. Cells were cultured overnight and then treated with 5 μM actinomycin D (Sigma-Aldrich, A1410) for 0 h, 2 h, 3 h, and 4 h. For RT-qPCR analysis, total RNAs were isolated and subjected to reverse transcription, and the mRNA levels of genes of interest were detected by RT-qPCR.

#### Puromycin assays (surface sensing of translation [SUnSET])

To assess global protein synthesis under Angiotensin II (AngII, 1 μM, 24 h), Puromycin was added to the culture medium at a final concentration of 1 μg/mL for 30 min at 37 °C before harvesting. To halt translation elongation, cells were treated with cycloheximide (10 μg/mL) for 15 min prior to harvesting. Cells were washed twice with ice-cold PBS and lysed on ice using RIPA buffer. Western blot was then performed using an anti-puromycin antibody.

### Quantification and statistical analysis

#### Statistical analysis

Quantitative results are presented as mean ± SD. One-way ANOVA was used for comparisons among groups, followed by Bonferroni post hoc tests. Statistical significance was set at *p* < 0.05, and analyses were performed using GraphPad Prism 4 (La Jolla, CA, USA).

## Supplemental information

Document S1. Figures S1–S3

Data S1. Original blots
