# Supplementary material for: N6-methyladenosine (m6A) modification of TXNIP in 3′UTR instigates abdominal aorta aneurysm in mice
Source: iScience. 2026 Jan 7;29(2):114630. doi: 10.1016/j.isci.2026.114630 (PMC12856352; doi:10.1016/j.isci.2026.114630)
Supplement: Document S1. Figures S1–S3 [file mmc1.pdf]

iScience, Volume *29*

Supplemental information

N6-methyladenosine (m^6A) modification of TXNIP in 3′UTR instigates abdominal aorta aneurysm in mice

Fransky Hantelys, Wenfeng Yin, and Ming Hui Zou

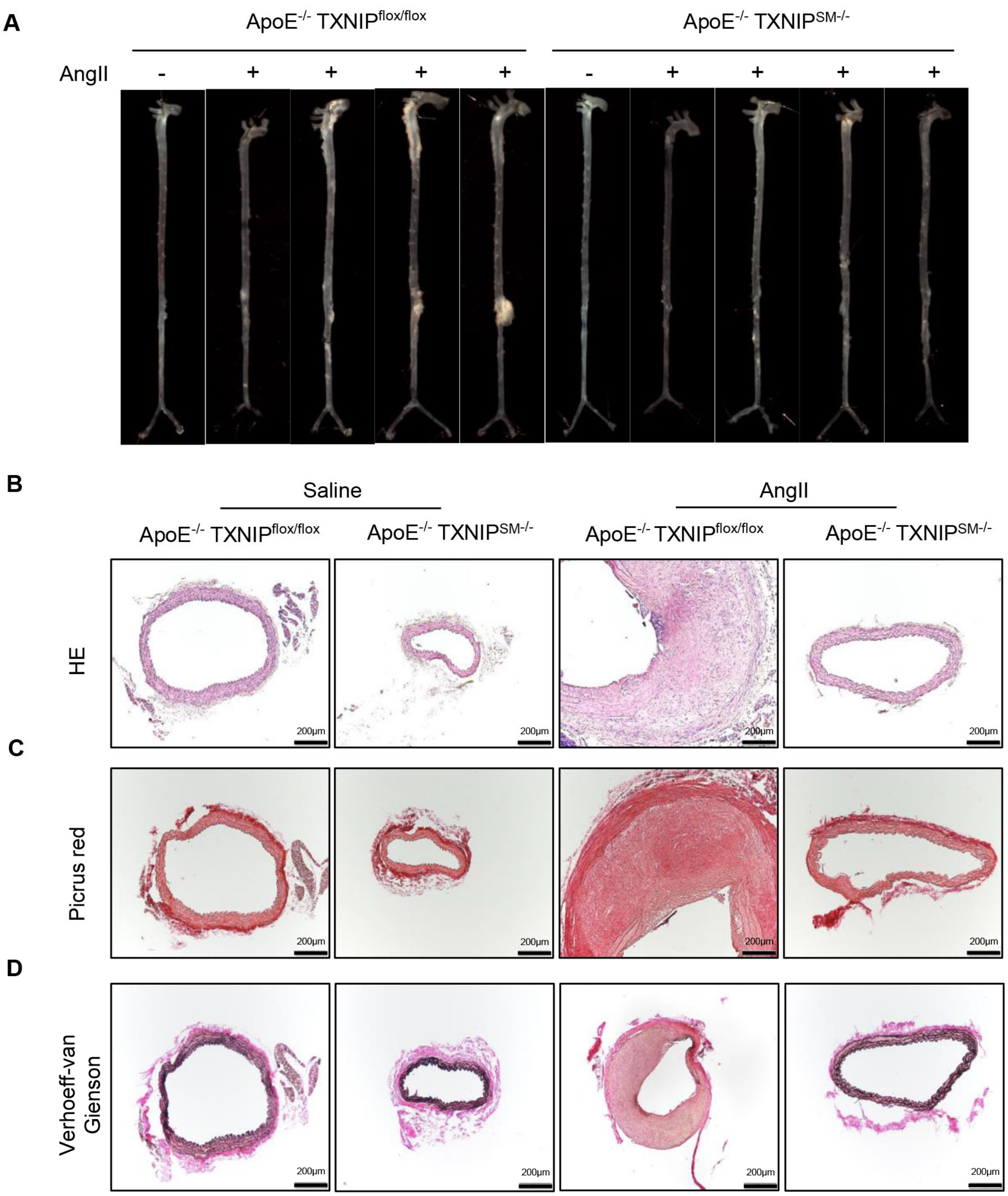

Supplemental Figure 1. Histology analysis of mouse aorta mages of suprarenal aortas of *ApoE⁻/⁻ TXNIP*$^{flox/flox}$ mice and *ApoE⁻/⁻ TXNIP*$^{SM-/-}$ mice.

(A) Representative images of suprarenal aortas of *ApoE*$^{-/-}$ *TXNIP*$^{flox/flox}$ mice and *ApoE*$^{-/-}$ *TXNIP*$^{SM-/-}$ mice with or without AngII infusion.

(B) Representative Hematoxylin and Eosin (H&E) staining.

(C and D) Representative Picrus red staining and Verhoeff-van Gienson staining for each group of mice (n = 5-22 mice/group).

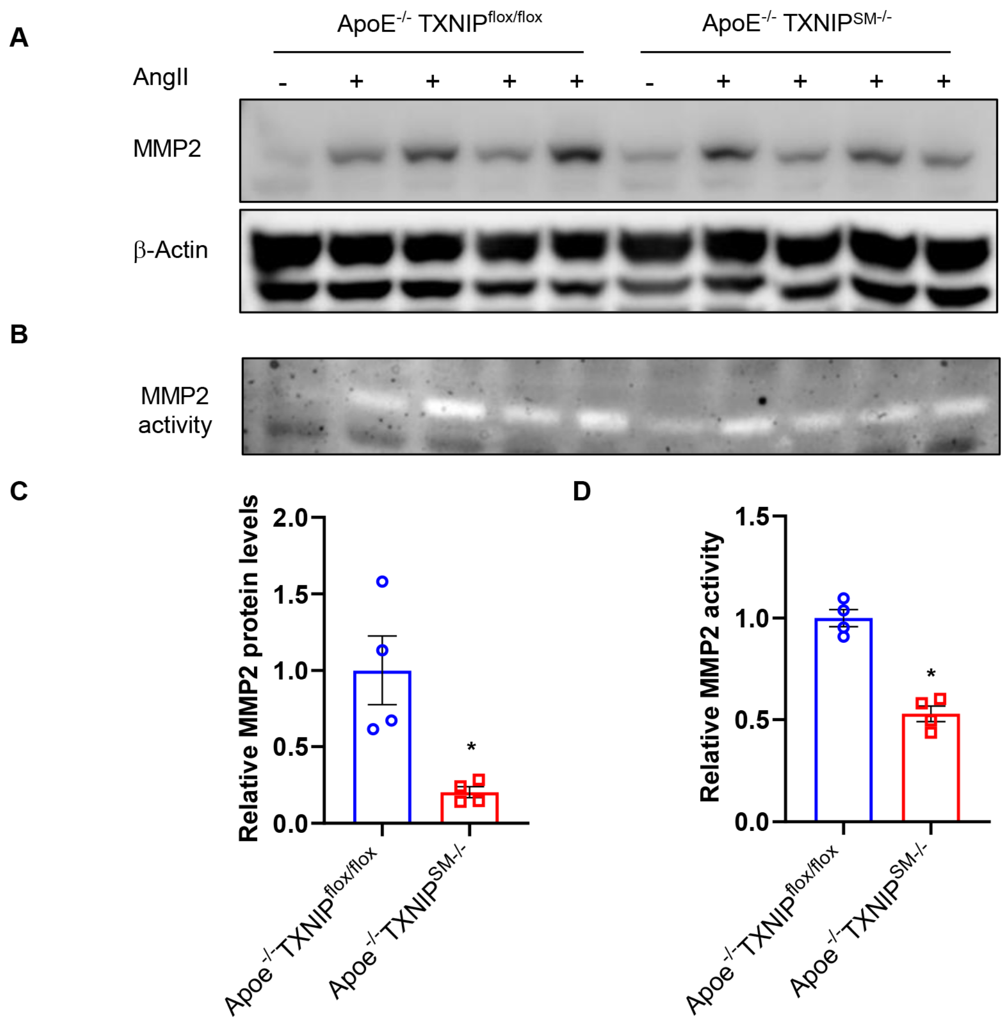

Supplemental Figure 2. AngII infusion caused higher expression and activity of matrix metaprotein (MMP2) in *ApoE$^{-/-}$ TXNIP$^{flox/flox}$* mice than their counterparts of *ApoE$^{-/-}$ TXNIP$^{SM-/-}$* mice *in vivo*.

(A) Western blot for MMP2 expression in the mouse aorta mages of suprarenal aortas of *ApoE$^{-/-}$ TXNIP$^{flox/flox}$* mice and *ApoE$^{-/-}$ TXNIP$^{SM-/-}$* mice infused with saline or AngII (1.44mg/kg/day) using an osmotic mini-pump for 28 days.

(B) Gelatin Zymography of MMP-2 activity in the mouse aorta mages of suprarenal aortas of *ApoE$^{-/-}$ TXNIP$^{flox/flox}$* mice and *ApoE$^{-/-}$ TXNIP$^{SM-/-}$* mice infused with saline or AngII (1.44mg/kg/day) using an osmotic mini-pump for 28 days.

(C and D) Quantification of MMP2 protein expression levels and MMP2 activity in the mouse aorta mages of suprarenal aortas of *ApoE$^{-/-}$ TXNIP$^{flox/flox}$* mice and *ApoE$^{-/-}$ TXNIP$^{SM-/-}$* mice infused with AngII (1.44mg/kg/day) using an osmotic mini-pump for 28 days. Values are represented as mean ± SD, * $p < 0.05$.

Supplemental Figure 3

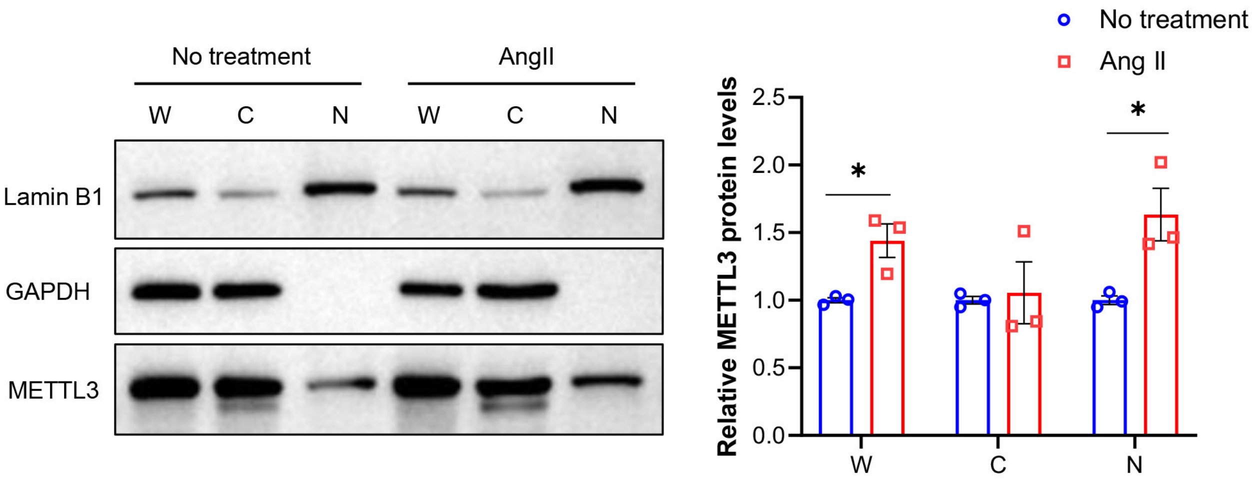

Supplemental Figure 3. Exposure of human aortic vascular smooth muscle cells (hASMCs) to AngII increases METTL3 nuclear translocation. Representative Western blot and protein quantification for AngII-induced nucleus relocation of METTL3 in cultured hASMCs. LaminB1: nuclear protein marker; GAPDH: cytosol protein marker. **W**: whole cell lysates; **C**: cytosol fractions; **N:** nuclear fractions. Values are represented as mean ± SD, * $p < 0.05$.
