# Supplementary material for: N6-methyladenosine (m6A) modification of TXNIP in 3′UTR instigates abdominal aorta aneurysm in mice
Source: iScience. 2026 Jan 7;29(2):114630. doi: 10.1016/j.isci.2026.114630 (PMC12856352; doi:10.1016/j.isci.2026.114630)

Figure 1B

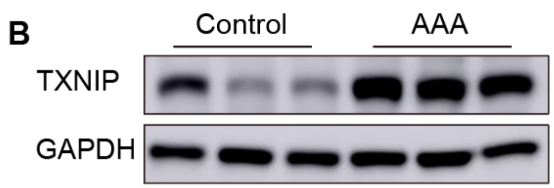

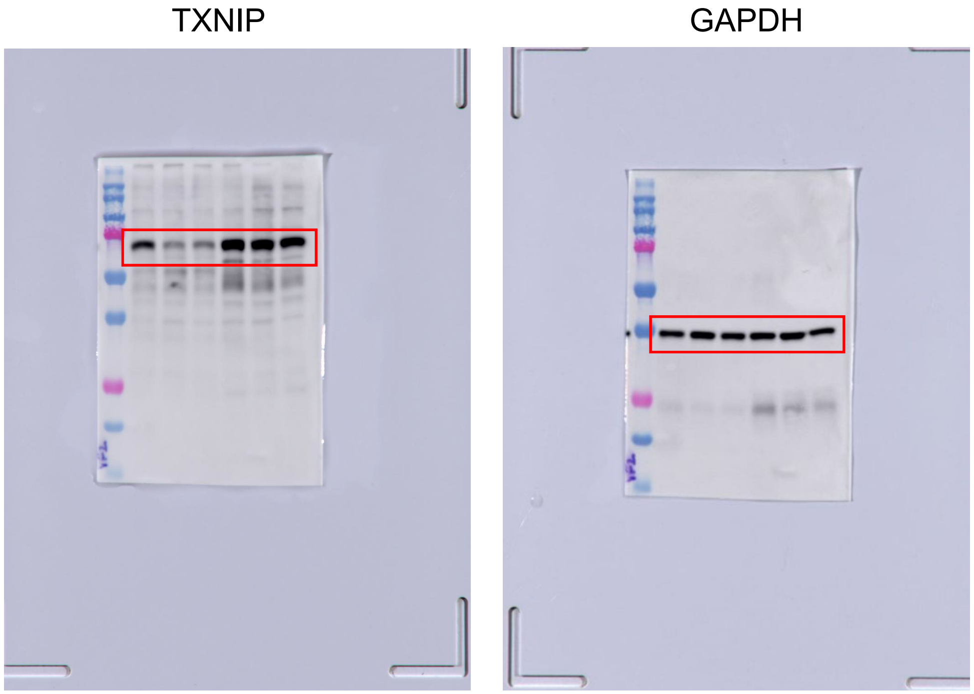

Figure 1I

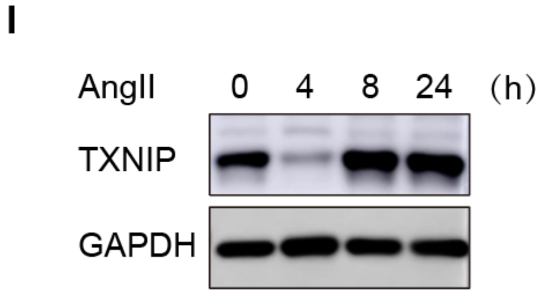

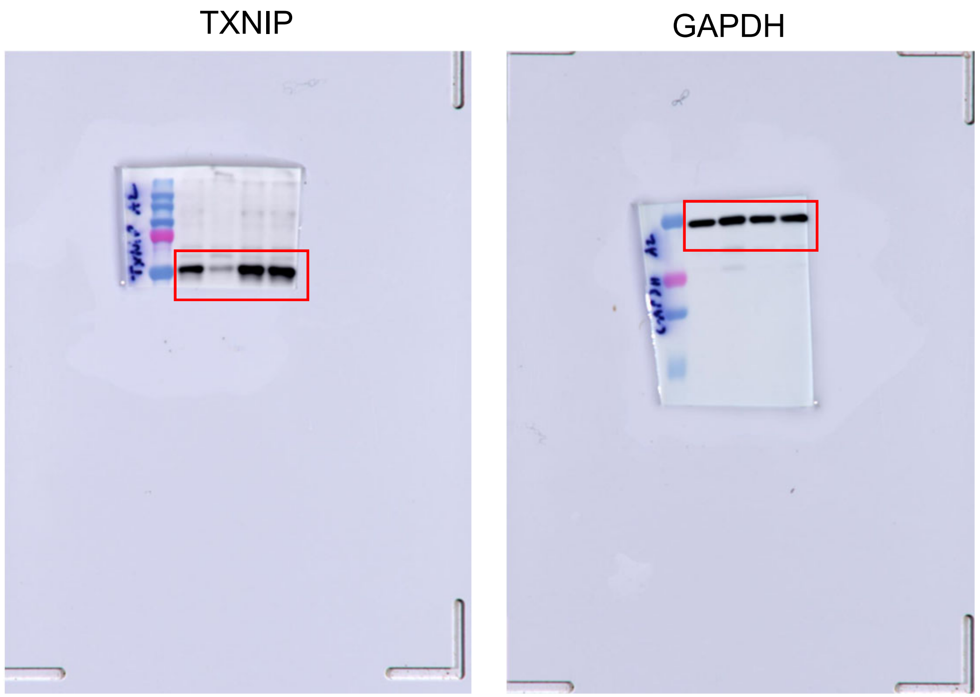

Figure 1L

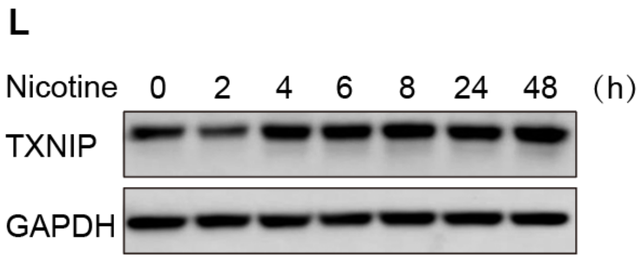

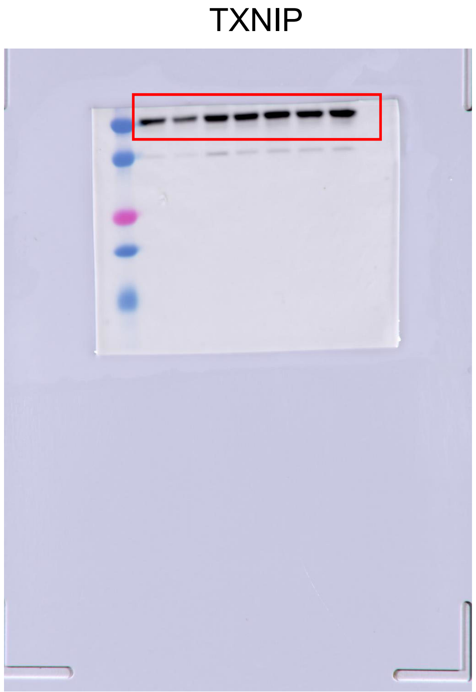

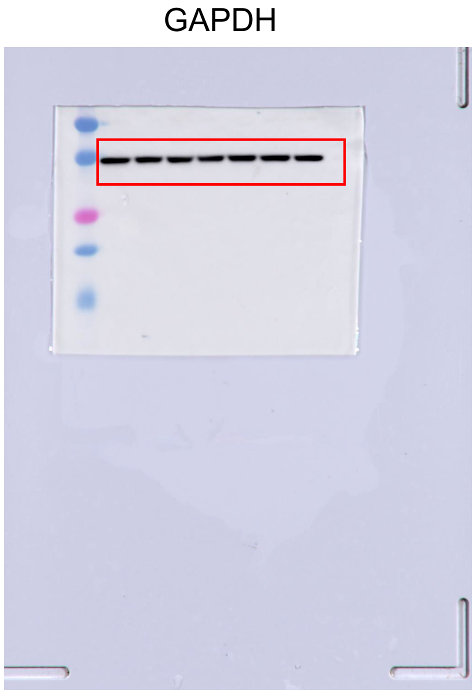

Figure 3C

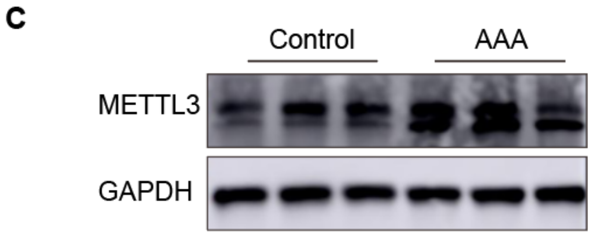

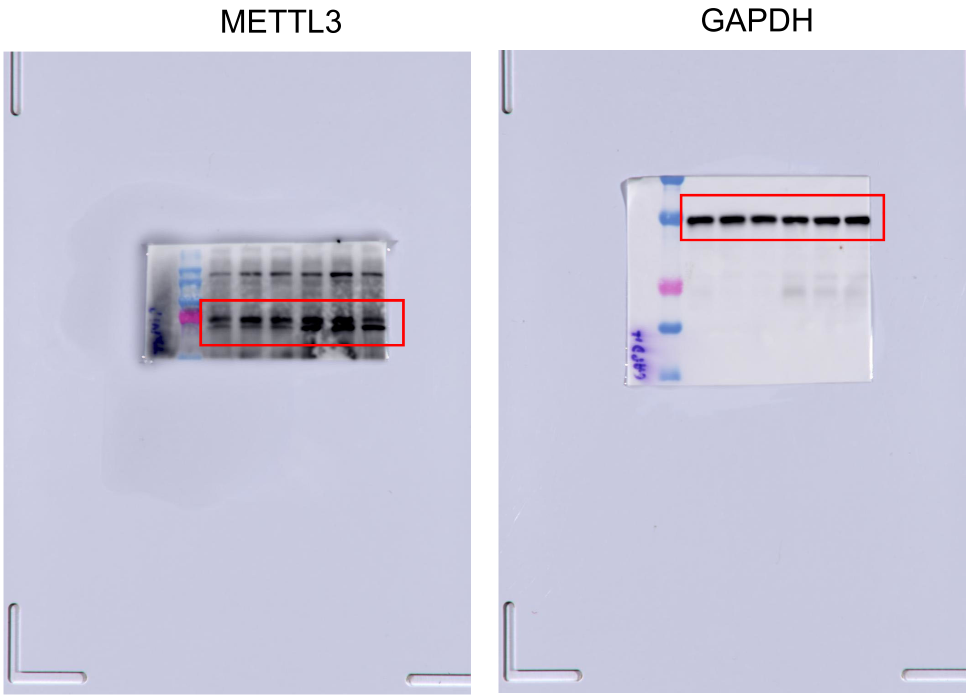

Figure 3D

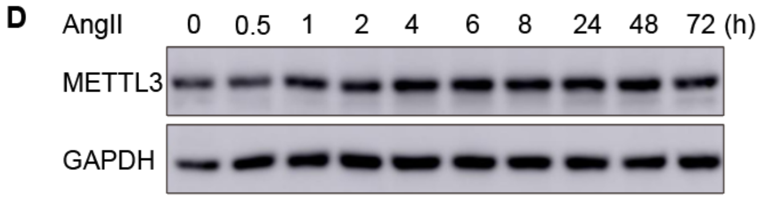

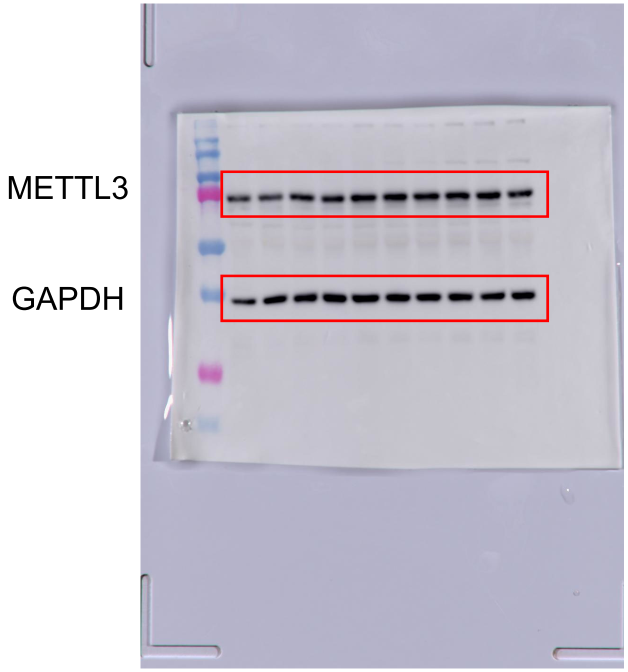

Figure 3E

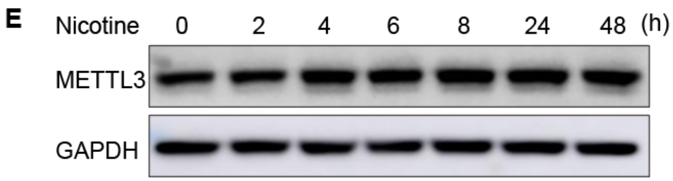

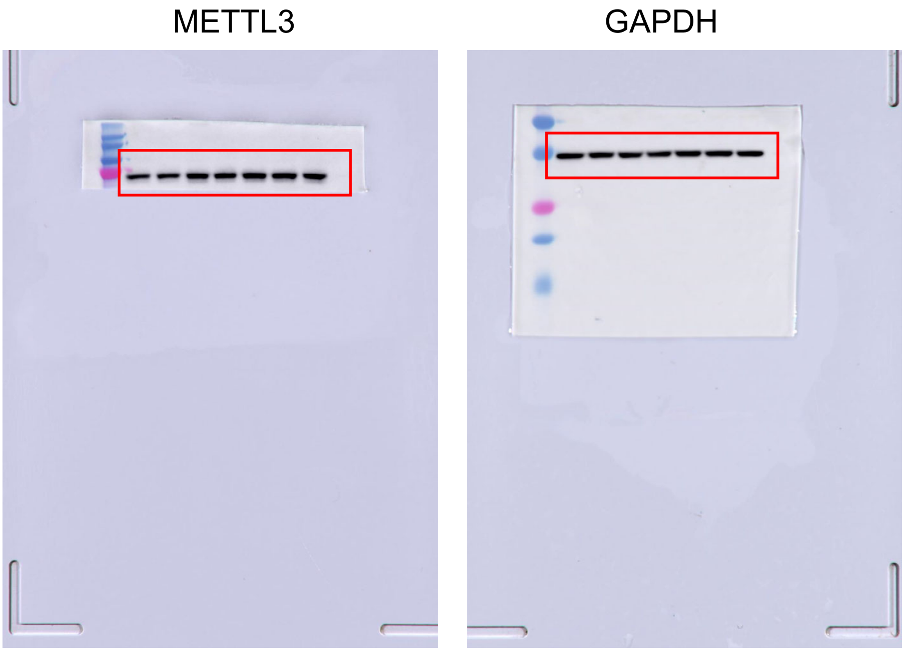

Figure 3F

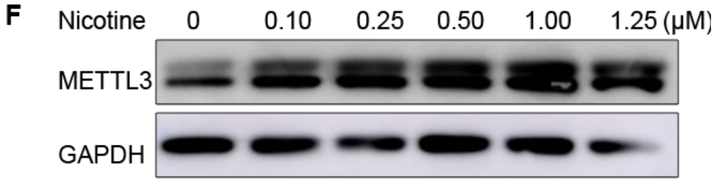

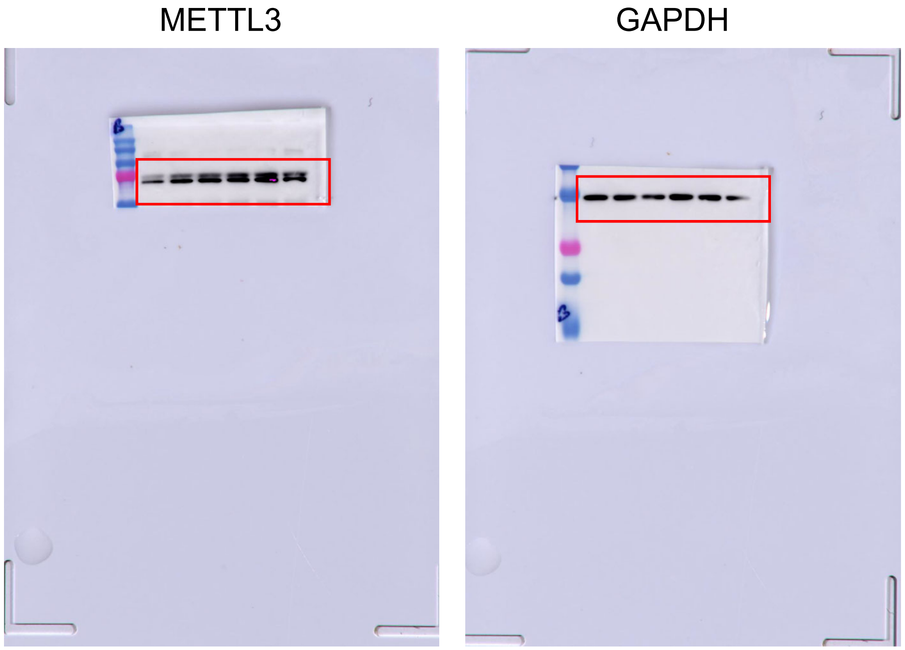

Figure 4A

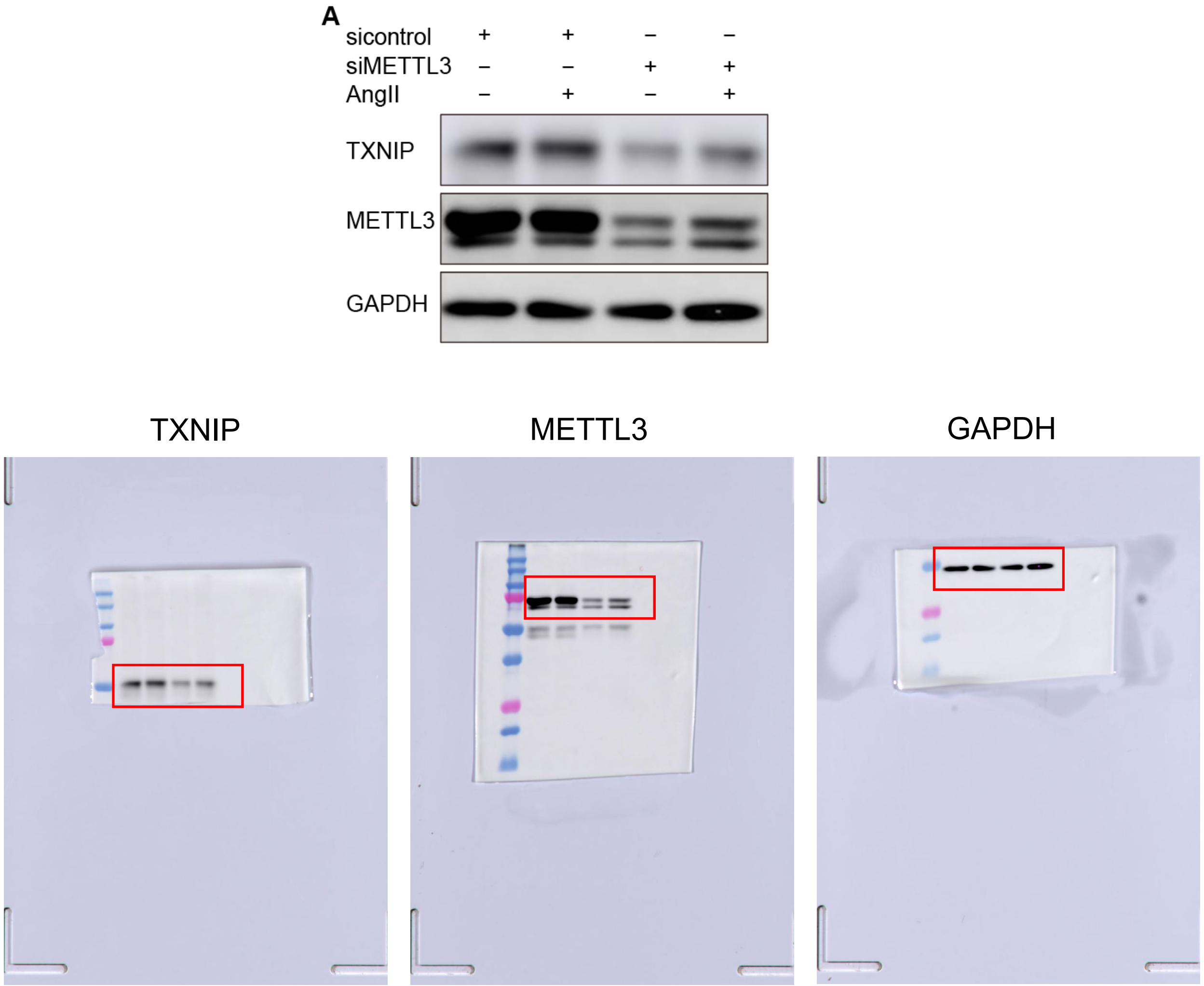
A
sicontrol + + – –
siMETTL3 – – + +
AngII – + – +
TXNIP
METTL3
GAPDH
TXNIP
METTL3
GAPDH

Figure 4B

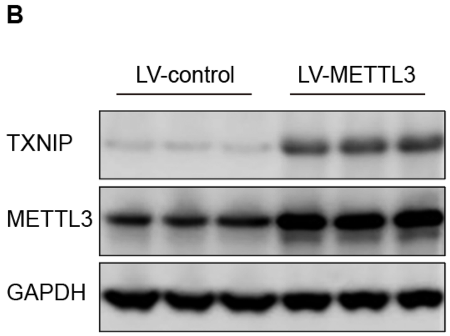
B
LV-control
LV-METTL3
TXNIP
METTL3
GAPDH

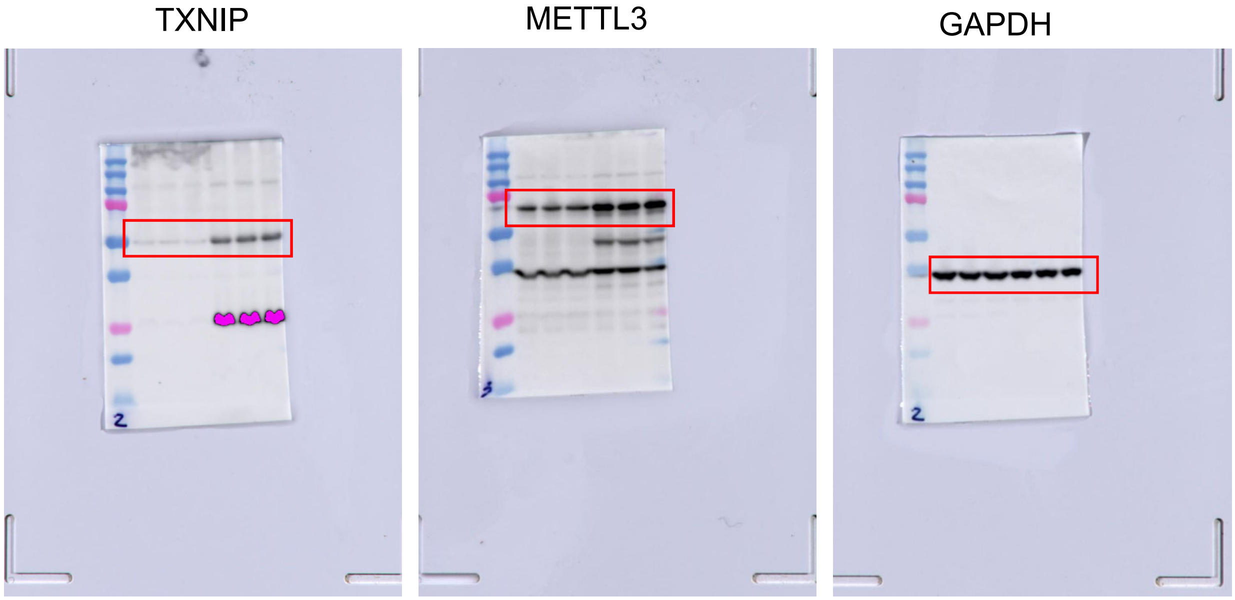
TXNIP
METTL3
GAPDH

Figure 5A

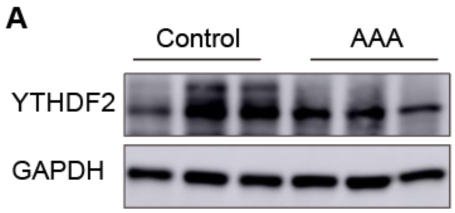

YTHDF2 GAPDH

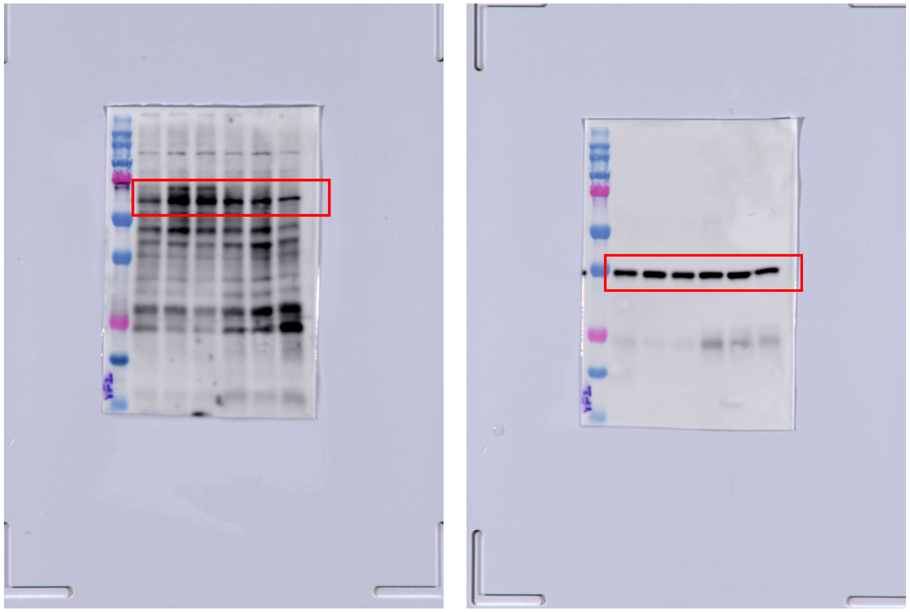

Figure 5B

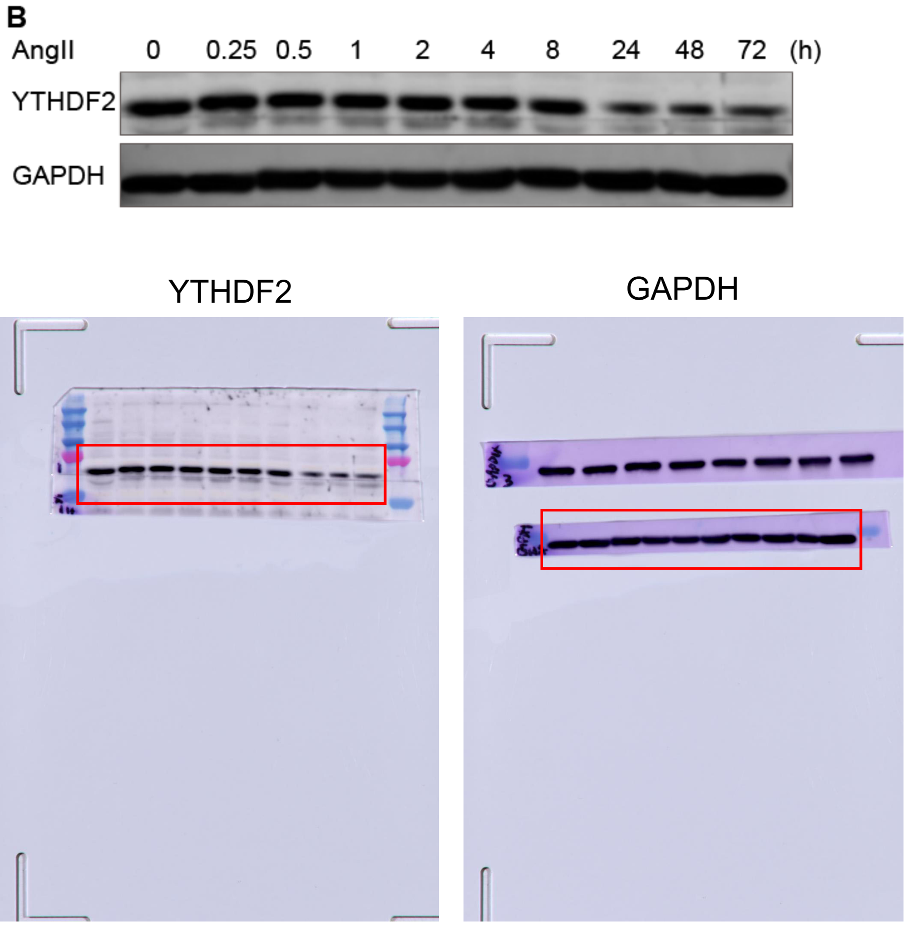

Figure 5C

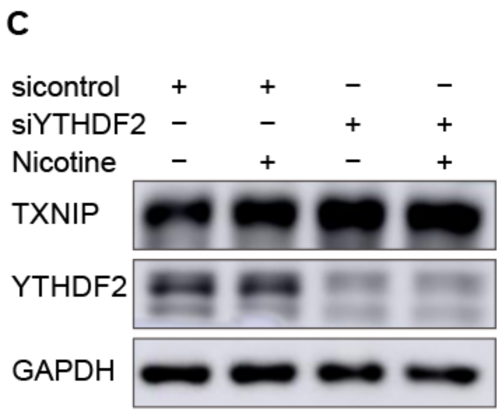

C
sicontrol + + − −
siYTHDF2 − − + +
Nicotine − + − +
TXNIP
YTHDF2
GAPDH

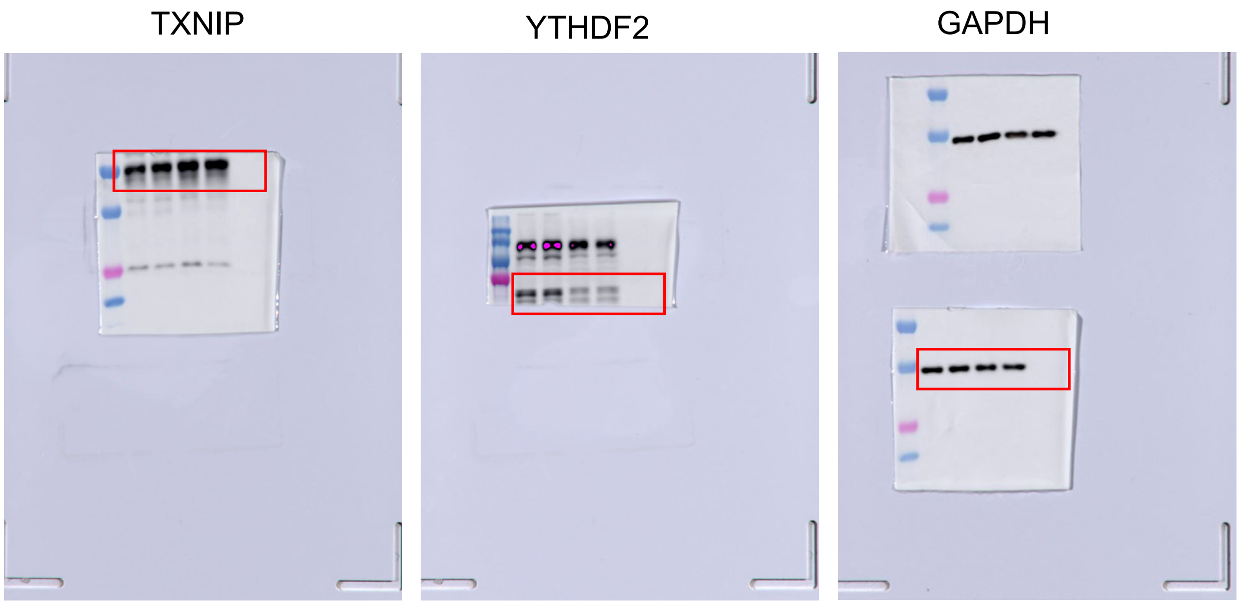

TXNIP
YTHDF2
GAPDH

Figure 6A

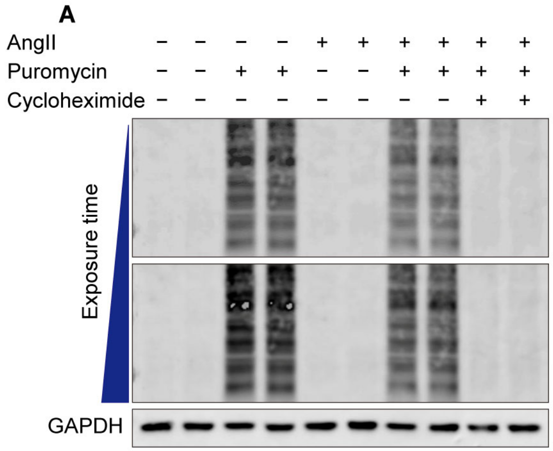
A
AngII − − − − + + + + + +
Puromycin − − + + − − + + + +
Cycloheximide − − − − − − − − + +
Exposure time
GAPDH

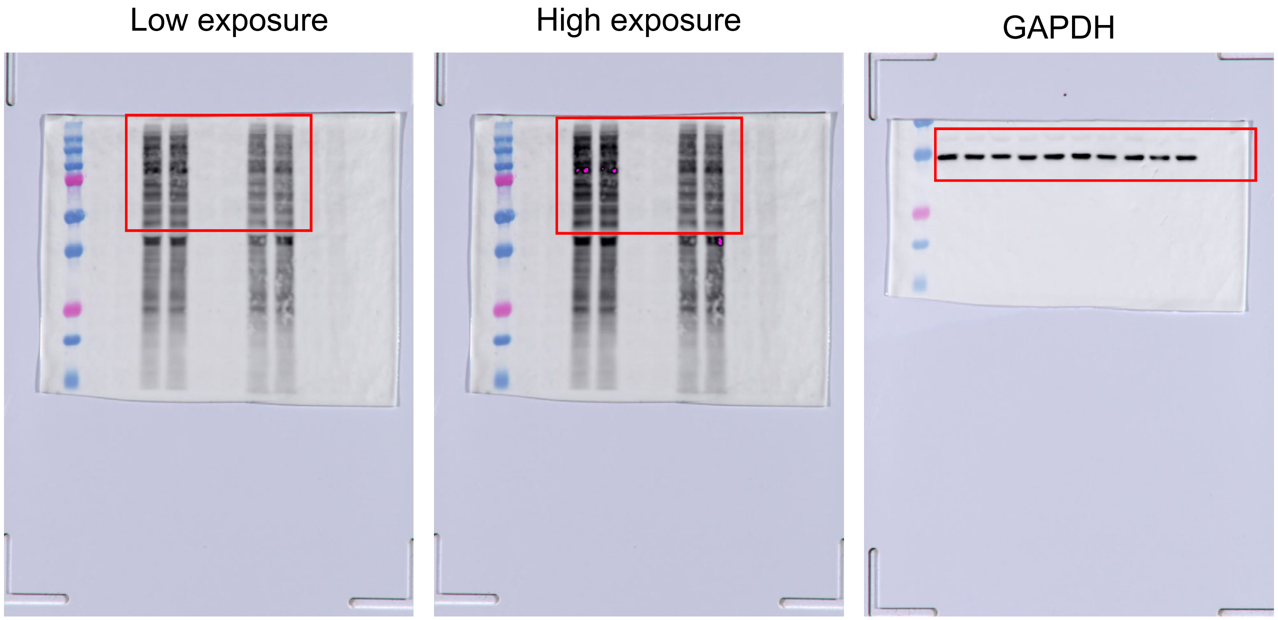
Low exposure
High exposure
GAPDH

Figure 6B

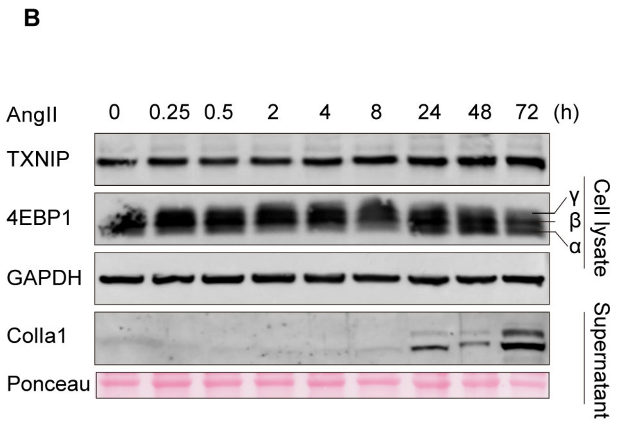
B
AngII
0
0.25
0.5
2
4
8
24
48
72
(h)
TXNIP
4EBP1
GAPDH
Colla1
Ponceau
γ
β
α
Cell lysate
Supernatant

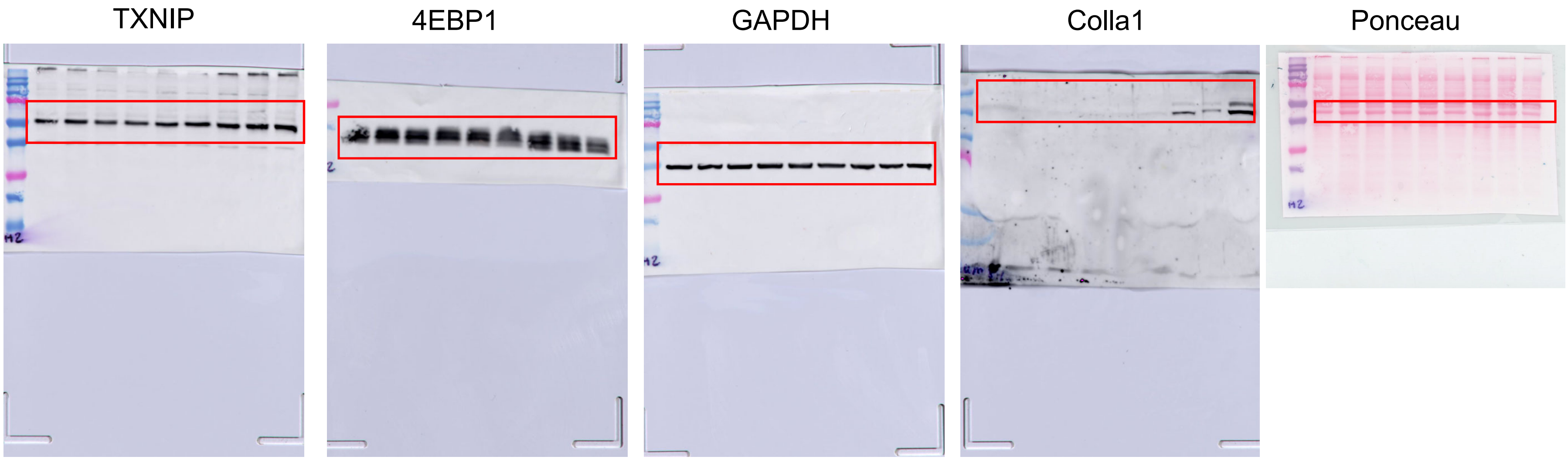
TXNIP
4EBP1
GAPDH
Colla1
Ponceau

Figure 6C

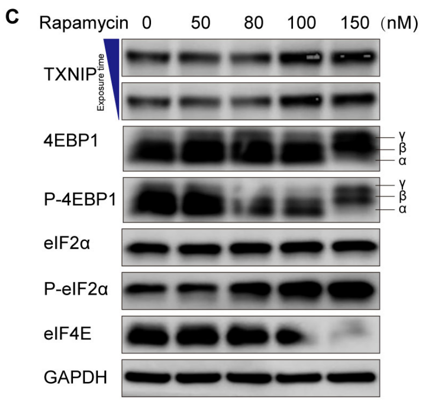
C
Rapamycin 0 50 80 100 150 (nM)
TXNIP
Exposure time
4EBP1
γ
β
α
P-4EBP1
γ
β
α
eIF2α
P-eIF2α
eIF4E
GAPDH

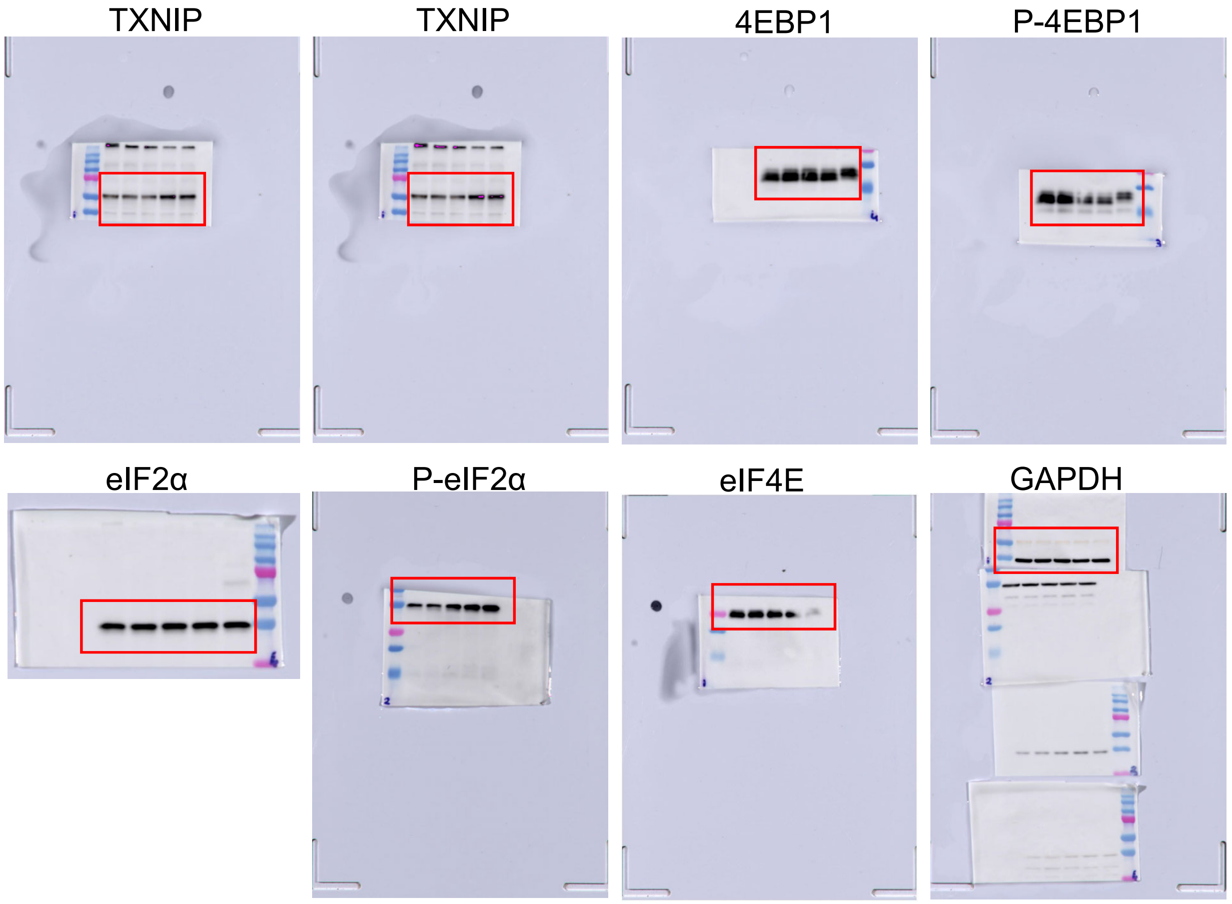
TXNIP
TXNIP
4EBP1
P-4EBP1
eIF2α
P-eIF2α
eIF4E
GAPDH

Figure 6D

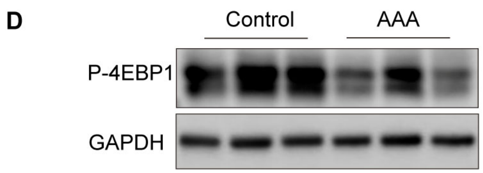

P-4EBP1

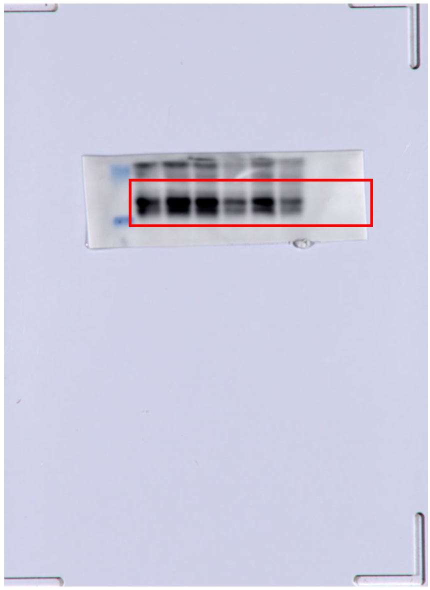

GAPDH

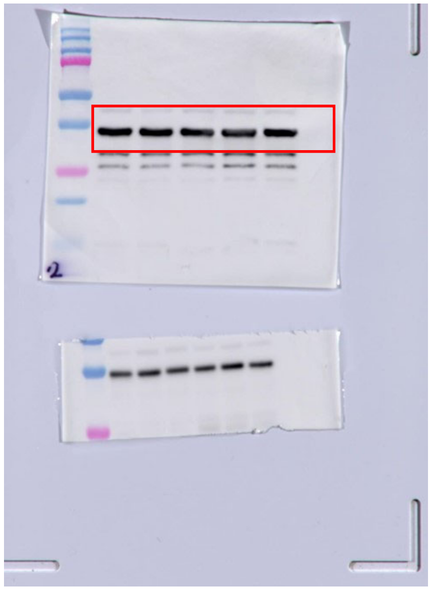

Figure 6E

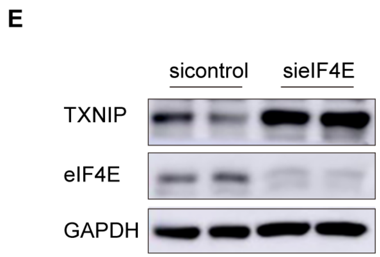

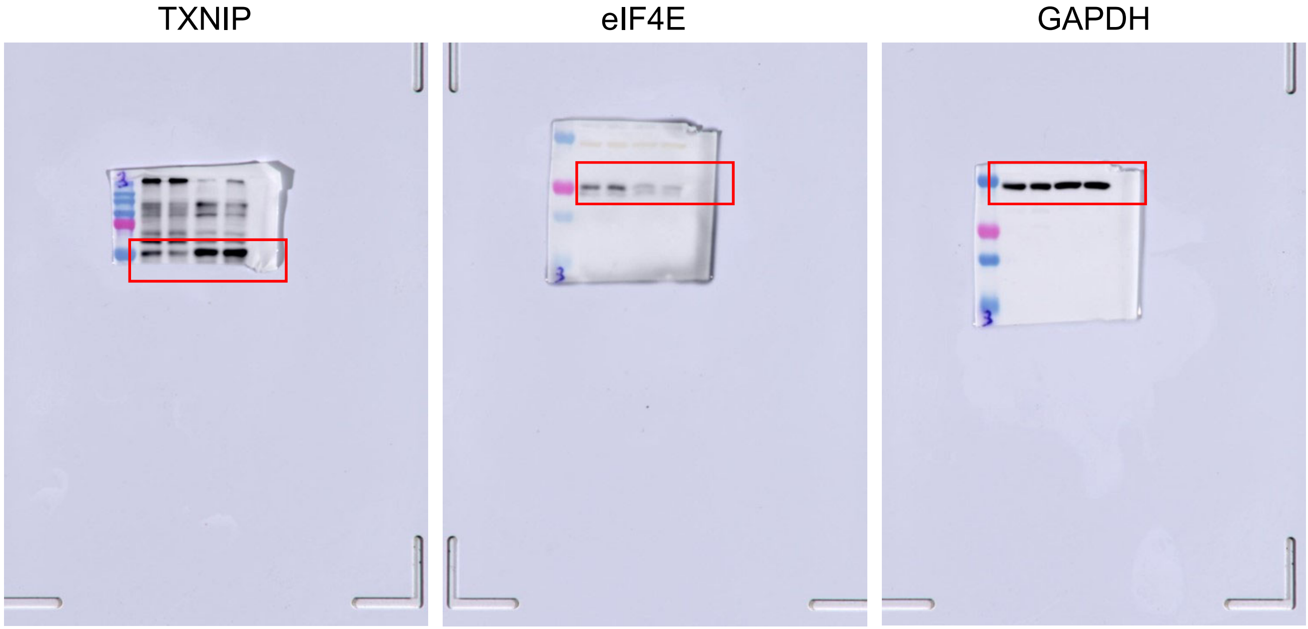

Figure 7A

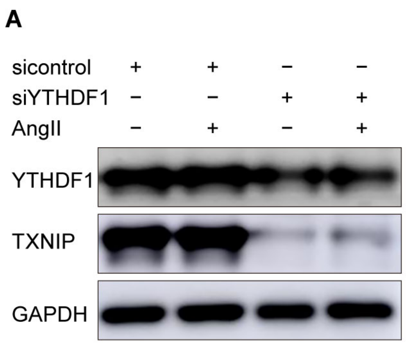
A
sicontrol + + − −
siYTHDF1 − − + +
AngII − + − +
YTHDF1
TXNIP
GAPDH

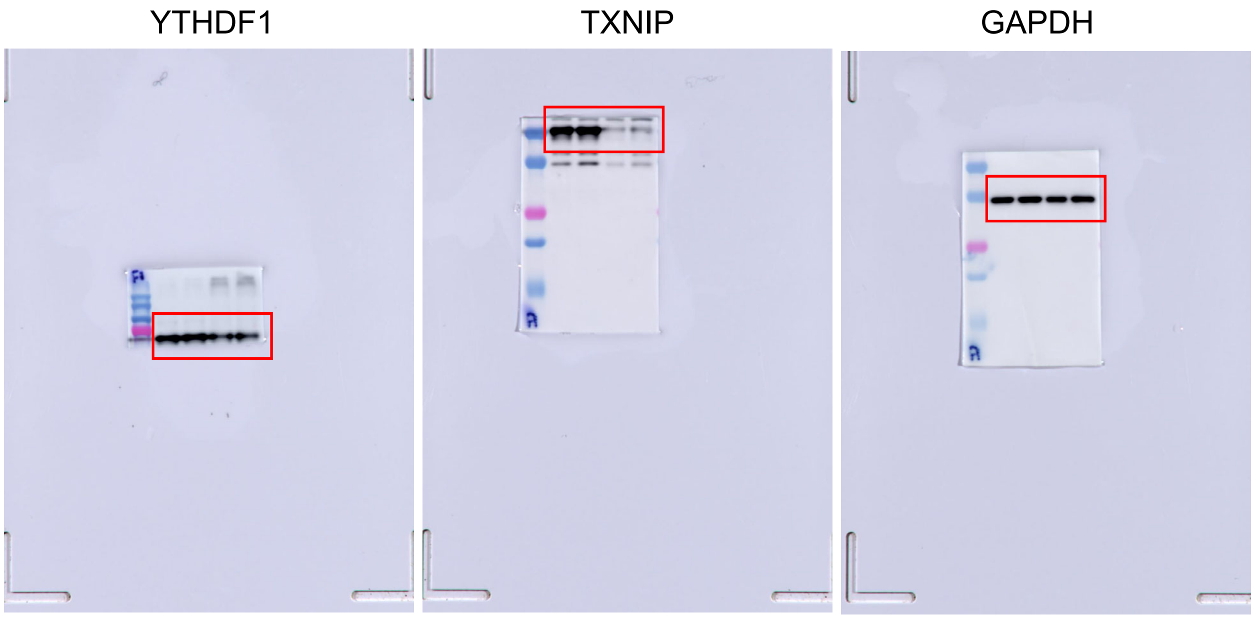
YTHDF1
TXNIP
GAPDH

Figure 7D

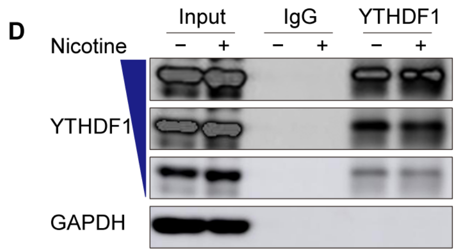

D
Input
IgG
YTHDF1
Nicotine
− + − + − +
YTHDF1
GAPDH

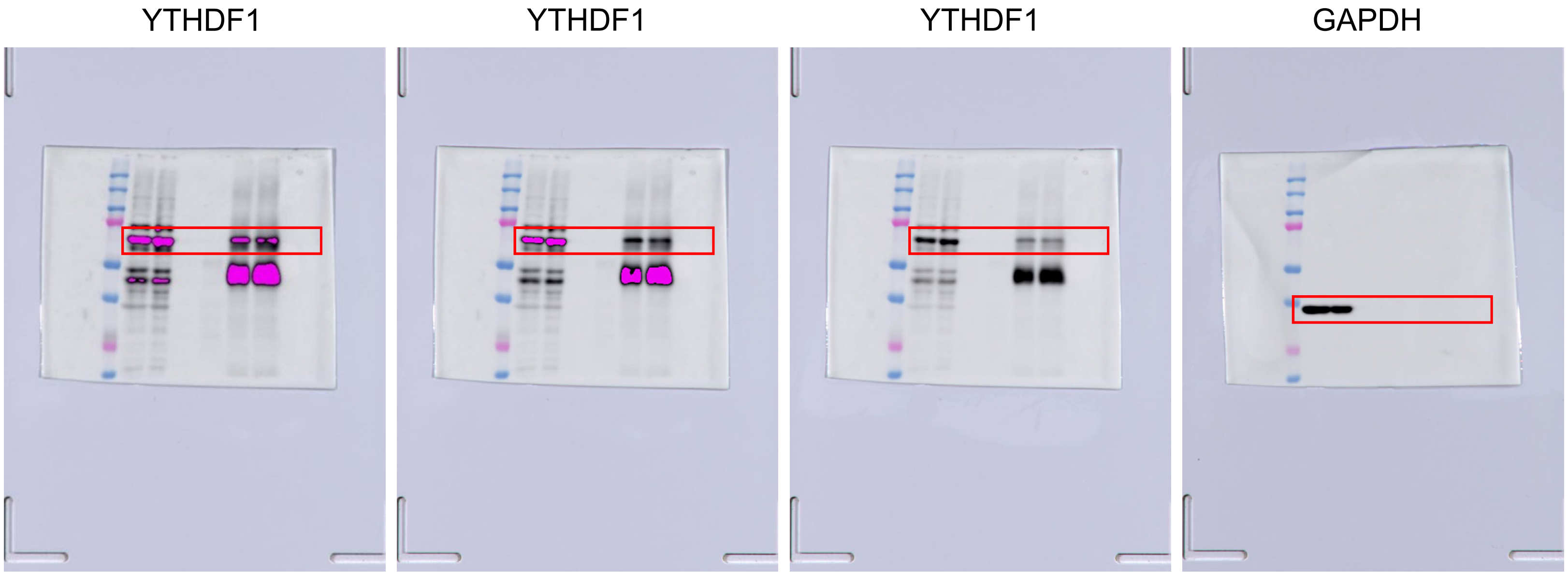

YTHDF1
YTHDF1
YTHDF1
GAPDH

Supplemental Figure 2A, 2B

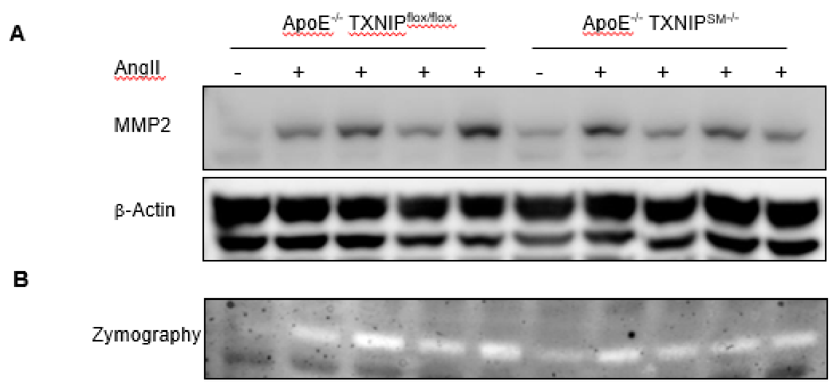

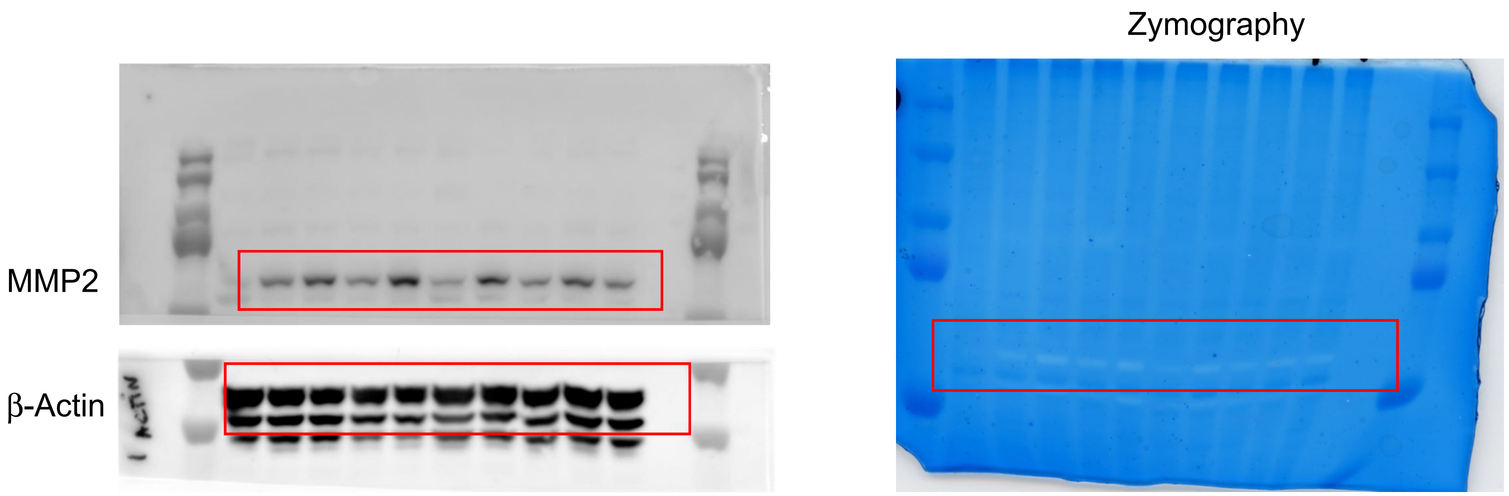

Supplemental Figure 3

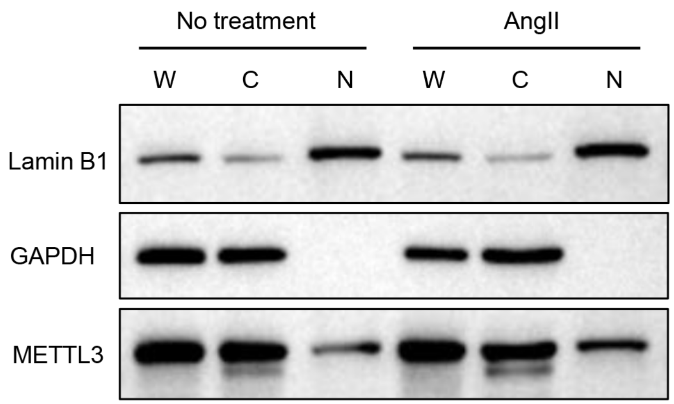

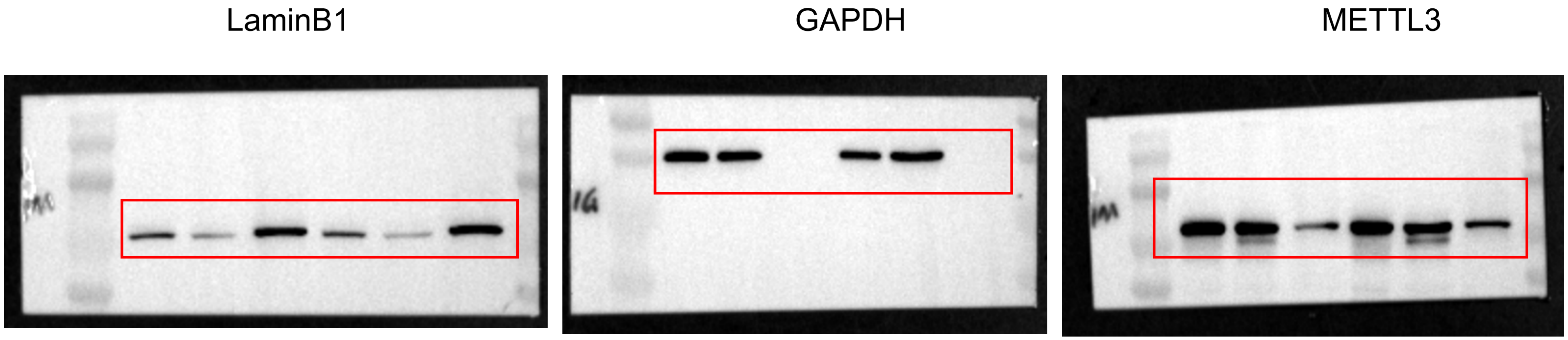

Supplement: Data S1. Original blots [file mmc2.pdf]
